# Mapping QTLs for PHS resistance and development of a deep learning model to measure PHS rate in *japonica* rice

**DOI:** 10.1002/tpg2.70109

**Published:** 2025-08-30

**Authors:** Soojin Jun, Mi Hyun Cho, Hyoja Oh, Younguk Kim, Dong Kyung Yoon, Myeongjin Kang, Hwayoung Kim, Seon‐Hwa Bae, Song Lim Kim, Jeongho Baek, HwangWeon Jeong, Jae Il Lyu, Gang‐Seob Lee, Changsoo Kim, Hyeonso Ji

**Affiliations:** ^1^ Department of Agricultural Biotechnology National Institute of Agricultural Sciences Jeonju Republic of Korea; ^2^ Department of Crop Science Chungnam National University Daejeon Republic of Korea; ^3^ Department of Computer Information Engineering Kunsan National University Gunsan Republic of Korea; ^4^ Department of Horticultural Bioscience Pusan National University Miryang Republic of Korea; ^5^ Fruit Research Division National Institute of Horticultural and Herbal Science (NIHHS) Wanju Republic of Korea; ^6^ Coastal Agriculture Research Institute Kyungpook National University Daegu Republic of Korea

## Abstract

Rice (*Oryza sativa* L.) is a staple food for more than half of the global population. Preharvest sprouting (PHS), which reduces yield and grain quality, presents a major challenge for rice production. The development of PHS‐resistant varieties is a major goal in *japonica* rice breeding. A deep learning model to automate PHS rate measurement was developed using the YOLOv8 algorithm. The model had high mean average precision (0.974). PHS rate measurements made using the model correlated strongly with manual measurements (*R* ^2^  =  0.9567). A population of 182 F_8_ recombinant inbred lines (RILs) was derived from a cross between the *japonica* rice cultivars, Junam and Nampyeong. The RIL genotypes at 763 single nucleotide polymorphism markers were determined using a rice target capture sequencing system and used to create a genetic map. The RILs were cultivated in the field (summer season) and the greenhouse (winter season) and their PHS rates were measured in both environments. Quantitative trait loci (QTLs) associated with PHS were present on chromosomes 3, 6, and 7 in the field, and on chromosomes 1, 2, 3, 6, 7, 8, and 11 in the greenhouse. Three QTLs on chromosomes 3, 6, and 7 showed stable effects in both environments. A search for candidate genes in the QTL *qPHS6* identified *Os06g0317200*. This gene encodes a glycine‐rich protein resembling *qLTG3‐1*, which controls PHS. The QTLs identified in this study and the deep learning model developed for measuring PHS rates will accelerate the development of rice varieties with enhanced resistance to PHS.

## Core Ideas

- Stable quantitative trait loci (QTLs) for preharvest sprouting (PHS) resistance were found on chromosomes 3, 6, and 7.
- The *Os06g0317200*, which encodes a glycine‐rich protein, was selected as a candidate gene for the major QTL *qPHS6* on chromosome 6.
- A YOLOv8‐based deep learning model was developed for rice PHS rate measurement.
- The deep learning model achieved high precision and PHS rate measurements made using the model correlated strongly with manual measurements (*R* ^2^ = 0.9567).

## Plain Language Summary

Preharvest sprouting (PHS) occurs when seeds germinate on the panicle prior to harvest before harvest due to high humidity and temperature. PHS can reduce crop quality and yield. To understand the genetic basis of PHS resistance in *japonica* rice, we performed quantitative trait locus (QTL) analysis using a population of recombinant inbred lines derived from a cross between two *japonica* rice cultivars with different PHS resistance levels. Three QTLs related to PHS with stable effects in different environments were identified on chromosomes 3, 6, and 7. To improve phenotyping efficiency, we developed a deep learning model to measure PHS rates automatically from seed images.

**Abbreviations**
ABA: abscisic acid
AI: artificial intelligence
dCAPS: derived cleaved amplified polymorphic sequence
GA: gibberellin
GRP: glycine‐rich protein
LOD: logarithm of odds
mAP: mean average precision
PHS: preharvest sprouting
QTL: quantitative trait locus
RIL: recombinant inbred line
SNP: single nucleotide polymorphism
YOLO: you only look once

## 1 INTRODUCTION

Rice (*Oryza sativa* L.) is a staple food for more than half of the global population (Bandumula, 2018) and provides sustenance to more than 3.5 billion people worldwide (Li et al., 2018). About 90% of the world's rice is produced and consumed in Asia, making this region critical to global food security (Singh et al., 2011). Despite advancements in agricultural practices, rice cultivation faces numerous challenges that threaten yield and grain quality. Preharvest sprouting (PHS) has emerged as a significant problem affecting rice production. PHS occurs when seeds germinate on the panicle prior to harvest, and is often due to environmental factors that disrupt seed dormancy mechanisms (Groos et al., 2002). Climate change has led to more unpredictable weather and longer periods of rain and high humidity during the rice maturation period, causing an increase in PHS incidence (Sohn et al., 2021). PHS reduces total starch content, resulting in lower grain quality and significant economic losses (Gubler et al., 2005; Zhang et al., 2020).

PHS is a complex quantitative trait that is controlled by multiple genes and influenced by environmental conditions (Vetch et al., 2019). Such polygenic traits present a challenge in breeding programs because many genetic factors contribute small effects (Mackay et al., 2009). This genetic complexity makes it difficult to develop resistant varieties, as multiple genes must be considered together. In addition, genotype–environment (G × E) interactions have a significant influence on PHS resistance, resulting in variable responses within genotypes across different environmental conditions (El‐Soda et al., 2014; He et al., 2014). These interactions pose an additional challenge to breeding programs, as the effects of genetic factors must be evaluated across multiple environments to identify stable and productive PHS‐resistant cultivars.

Various factors, including the regulation of seed dormancy and germination by phytohormones, influence the induction of PHS in rice (Shu et al., 2016). Abscisic acid (ABA) is a key hormone that promotes seed dormancy by inhibiting germination (Chen et al., 2021). In rice, the *9‐cis‐epoxycarotenoid dioxygenase* (*OsNCED*) and *ABA 8ʹ‐hydroxylase* (*OsABA8ʹox*) genes encode major regulators of ABA biosynthesis and their expression is essential for controlling ABA levels (Sohn et al., 2021; Suriyasak et al., 2020). NCED catalyzes the rate‐limiting step in the ABA biosynthesis pathway by converting 9‐cis‐epoxycarotenoids into xanthoxin, a key precursor of ABA (Xiong & Zhu, 2003). Conversely, ABA is catabolized by ABA 8ʹ‐hydroxylase into phaseic acid, which reduces ABA levels and thereby facilitates release from dormancy and germination (Sano & Marion‐Poll, 2021; Zhu et al., 2009). While ABA plays a central role in maintaining seed dormancy, the phytohormone gibberellin (GA) induces germination. GA is transported to the aleurone layer of the endosperm where it triggers α‐amylase expression and facilitates the hydrolysis of starch into sugars that supply energy for germination (Graeber et al., 2012; Kaneko et al., 2002). Environmental stress can disrupt the balance between ABA and GA, leading to PHS. High temperature, high humidity, and prolonged rainfall during the late stages of seed maturation increase the risk of PHS (H. Lee, Choi, et al., 2021). Physical factors also affect PHS; for instance, the seed coat delays germination by functioning as a physical barrier that regulates water and oxygen permeability (Sohn et al., 2021).

More than 185 quantitative trait loci (QTLs) related to PHS, seed dormancy, low‐temperature germination, and germination index have been identified in the rice genome (Sohn et al., 2021). However, QTL studies of this trait within *japonica* populations have been limited even though there is significant variation in PHS resistance between *japonica* rice cultivars (J.‐S. Lee, Chebotarov, et al., 2021). Recently, understanding of the genetic basis underlying PHS resistance in *japonica* rice has been advanced by the identification of key QTLs and candidate genes. Fujino et al. (2004) conducted a mapping study using backcross inbred lines (BILs), derived from a cross between the *japonica* varieties Italica Livorno and Hayamasari, to locate QTLs for germinability at low temperature. They found that *qLTG3‐1*, a QTL on chromosome 3, accounted for 35% of the phenotypic variation. Hori et al. (2010) used both BILs and chromosome segment substitution lines (CSSLs) derived from a cross between Nipponbare and Koshihikari to identify major QTLs for PHS resistance on chromosomes 3 and 12. They found that *qLTG3‐1* co‐localizes with PHS resistance traits. Mizuno et al. (2018) used CSSLs from a cross between Owarihatamochi and Koshihikari to identify two QTLs for seed dormancy, *qSDR9.1* and *qSDR9.2*, on chromosome 9 and demonstrated their roles in conferring PHS resistance. Cheon et al. (2020) employed recombinant inbred lines (RILs) from a cross between Odae and Unbong40. They found QTLs for PHS on chromosomes 3, 4, and 11, one of which, *qPHS‐11*, had consistent effects on PHS resistance in both field and greenhouse conditions. Jang et al. (2020) identified overlapping QTLs for PHS, seed dormancy, and low‐temperature germination on chromosome 1 in F_2:3_ populations derived from a cross between Jinsang and Gopum, which suggested pleiotropic effects between these traits. Jang et al. (2023) refined these findings further by fine mapping the QTL to a 50 kbp region of chromosome 1. This identified *Os01g0111600*, which encodes a regulator of ABA signaling‐mediated seed germination, as a likely candidate gene contributing to PHS resistance. Very recently, C.‐M. Lee et al. (2023) explored the genetic potential of *japonica* weedy rice in enhancing PHS resistance by conducting a QTL analysis of F_8_ RIL populations derived from a cross between a Korean *japonica* weedy rice line and Hwayeong. This study identified two stable QTLs, *qPH7* and *qPH2*, on chromosomes 7 and 2, respectively. *qPH7* was fine‐mapped to a 210‐kbp region containing *Os07g0584366*, a gene whose expression was significantly higher in resistant lines under PHS‐inducing conditions.

G.‐A. Lee et al. (2017) analyzed 144 *japonica* accessions in a genome‐wide association study and identified 10 single nucleotide polymorphisms (SNPs) that were significantly associated with PHS resistance. They developed a regression model showing an *R* ^2^ value of 0.401 in *japonica* rice. J.‐S. Lee, Chebotarov, et al. (2021) utilized a diverse panel of 277 rice accessions, including temperate and tropical *japonica* varieties, and detected two major loci on chromosomes 1 and 4 that were linked to ABA, GA, and auxin‐mediated signaling pathways, providing insights into the hormonal regulation of PHS resistance.

**Core Ideas**

- Stable quantitative trait loci (QTLs) for preharvest sprouting (PHS) resistance were found on chromosomes 3, 6, and 7.
- The *Os06g0317200*, which encodes a glycine‐rich protein, was selected as a candidate gene for the major QTL *qPHS6* on chromosome 6.
- A YOLOv8‐based deep learning model was developed for rice PHS rate measurement.
- The deep learning model achieved high precision and PHS rate measurements made using the model correlated strongly with manual measurements (*R* ^2^ = 0.9567).

Map‐based cloning studies have identified key genes associated with PHS resistance, including *qLTG3‐1*, *Seed Dormancy 4 (Sdr4*), *semidwarf 1–2* (*SD1‐2*), and *Seed Dormancy 6* (*SD6*) (Sohn et al., 2021). *qLTG3‐1* encodes a glycine‐rich protein (GRP) that induces vacuolization in the tissue covering the embryo, which weakens it by changing the structure of the cell wall. This is an important mechanism that promotes rice germination, even under low‐temperature conditions (Fujino et al., 2008). *SDR4* suppresses PHS and maintains seed dormancy in rice by repressing GA synthesis and regulating germination genes (Sugimoto et al., 2010 **;** B. Zhao et al., 2022). *SD1‐2* produces a dwarf phenotype by reducing plant height through the inhibition of GA biosynthesis; it also lowers GA accumulation in seeds, enhancing seed dormancy (Spielmeyer et al., 2002; Ye et al., 2015). *SD6* is a bHLH transcription factor that interacts antagonistically with *INDUCER OF CBF EXPRESSION 1* (*ICE2*). It promotes seed germination by activating *ABA8ʹox3* to decrease ABA levels and also indirectly inhibits *NCED2*, reducing ABA biosynthesis (Xu et al., 2022).

Efficient and accurate phenotyping methods that enable the rapid and precise assessment of traits such as PHS resistance across large populations are crucial for the development of breeding programs. Traditional phenotyping methods are often labor‐intensive and time‐consuming, and may not always produce accurate data (Furbank & Tester, 2011; Yang et al., 2020). Deep learning algorithms, a form of artificial intelligence (AI), can be particularly effective tools for rapid and precise trait analysis (Nabwire et al., 2021). “You only look once” (YOLO) is a powerful object detection framework known for its speed and accuracy (Jiang et al., 2022). A PHS measurement model using the YOLO algorithm that surmounted the limitations imposed by manual methods would enable efficient and accurate phenotyping by distinguishing between germinated and ungerminated seeds with high precision, even under conditions of high seed density. This automated approach would provide objective and consistent assessments, significantly reducing the effects of human error and variability.

We developed a deep learning model that incorporated the YOLOv8 algorithm and was capable of automated PHS rate measurement. In addition, we mapped QTLs associated with PHS resistance in a population of F_8_ RILs derived from a cross between the *japonica* cultivars Junam and Nampyeong. Our findings provide critical tools for the future breeding of PHS‐resistant rice varieties.

## 2 MATERIALS AND METHODS

### 2.1 Plant materials and growing conditions

The *japonica* rice cultivars, Junam and Nampyeong, were used in this study. Junam exhibits moderate susceptibility to PHS whereas Nampyeong is resistant to PHS. A population containing 182 F_8_ RILs was derived from a cross between Junam and Nampyeong. The RILs and their parental lines were cultivated in the field (2023) and under greenhouse (2024) conditions at the National Institute of Agricultural Sciences of the Rural Development Administration (Jeonju, Republic of Korea). In the field experiment, 20 seedlings per RIL were transplanted in a row. The daily mean temperature during the grain filling stage (from the heading date to harvest) ranged from 15°C to 30°C (Figure S1). In the greenhouse experiment, one seedling per RIL was transplanted into a 150 mm diameter pot. An appropriate fertilizer containing nitrogen, phosphorus, and potassium was applied twice at monthly intervals to ensure optimal plant growth. Plants in the greenhouse were maintained at a mean temperature of 23°C–26°C and at 41%–79% relative humidity (Figure S1).

### 2.2 Measurement of PHS rate

During the heading stage, three main panicles from three plants per RIL were randomly selected and labeled with the heading date. The labeled panicles were harvested 41 days after heading (DAH) in the field experiment and 40 DAH in the greenhouse experiment when the accumulated temperature after heading reached approximately 1000°C. Seeds from each panicle were placed on wet filter paper in 15‐cm Petri dishes. The dishes were incubated in a growth chamber at 25°C with 100% relative humidity for 5 days. Images of the seeds were captured after incubation using an RGB ILCE‐7RM3 digital camera (Sony) in an image acquisition box. The numbers of germinated and ungerminated seeds were counted manually. A seed was considered to have germinated if the radicle visibly protruded through the seed coat. Abnormal (i.e., damaged or diseased) seeds were excluded from the count.

The PHS rate was calculated using the following formula:

$$\begin{array}{ccc} & & \mathrm{PHS}\;\mathrm{rate}\;\left(\%\right)\\ & & \;=\left\{\frac{\mathrm{germinated}\;\mathrm{seeds}}{\mathrm{total}\;\mathrm{seeds}\;\left(\mathrm{germinated}\;\mathrm{seeds}+\mathrm{ungerminated}\;\mathrm{seeds}\right)}\right\}\\ & & \;\times \;100 \end{array}$$

### 2.3 Development of the deep learning model

**FIGURE 1:**
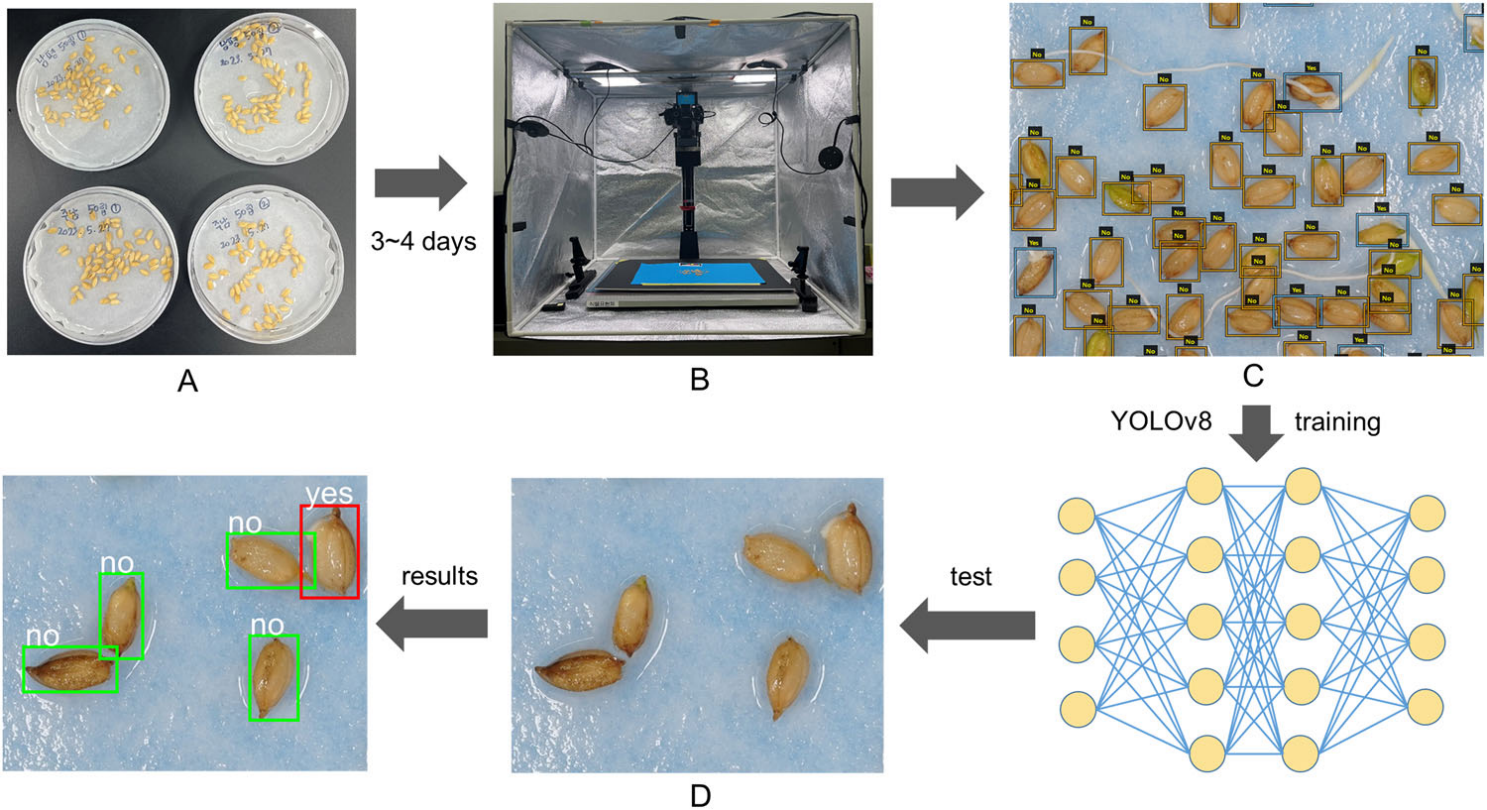
Development of a deep learning model for measuring the germination rate of rice seed. (A) Germination tests for the *japonica* varieties Junam and Nampyeong. (B) Box used in the acquisition of rice seed images. (C) Labeling of rice seeds using bounding boxes. (D) Analysis of germination with the YOLOv8 deep learning algorithm.

Samples of 100 seeds from the cultivars Junam, Nampyeong, and Hwayeong were placed in 15 cm Petri dishes and incubated at 25°C with 100% relative humidity. Images were captured after 3, 4, and 5 days of incubation with 10 replicates per day, resulting in a total of 9000 seed images. Additionally, approximately 14,000 images of seeds from a field‐based experiment with the RIL population derived from a cross between Junam and Nampyeong that exhibited varying degrees of PHS were added to the dataset. The VGG Image Annotator software (Dutta & Zisserman, 2019) was used to annotate the images with bounding boxes. Each seed in an image was classified as germinated (“yes”), ungerminated (“no”), or abnormal (Figure 1). The dataset was divided into training and test subsets with an 8:2 ratio. The YOLOv8 deep learning algorithm (https://www.ultralytics.com) was selected for its efficiency in object detection tasks (Diwan et al., 2023).

Model training was conducted within the Google Colab environment (https://colab.google/). Hyperparameters, such as image size, batch size, and the number of epochs, were adjusted to optimize model performance. The mean average precision (mAP) tended to converge on 1, while box loss, class loss, and distribution focal loss approached 0. Training was halted when no further improvement was observed as the epochs progressed. A confusion matrix was generated from the test data to assess model accuracy, including the metrics precision, recall, and F1‐score. Precision was the proportion of correctly identified positive instances out of all positive identifications, while recall denoted the proportion of correctly identified positive instances out of all actual positives. The F1‐score represented the harmonic mean of precision and recall (Hicks et al., 2022). These metrics were used to evaluate the model's performance in classifying seeds into the three categories (germinated, ungerminated, and abnormal).

After training, the deep learning model was applied to a dataset of new images from the greenhouse PHS experiment with the RIL population derived from a cross between Junam and Nampyeong. The model automatically detected and counted germinated seeds, and calculated the PHS rate in a manner analogous to the manual method. The PHS rates obtained using the deep learning model were compared with those from manual assessments. The relationship between the PHS rates determined using the two methods was assessed using Pearson's correlation coefficient; a high correlation coefficient indicated a strong agreement between methods.

### 2.4 Whole genome resequencing data analysis

The whole genome sequencing data of the parental varieties Junam and Nampyeong were obtained from previous studies (Cheon et al., 2018; Kang et al., 2019). The data were analyzed following the method described by Kumagai et al. (2019). Trimmomatic v0.36 (Bolger et al., 2014) was used to remove low‐quality bases and adapter sequences from the raw reads. The cleaned reads were aligned with the IRGSP‐1.0 Nipponbare reference genome (Kawahara et al., 2013) using BWA‐mem v0.7.12 (https://sourceforge.net/projects/bio‐bwa/). Polymerase chain reaction (PCR) duplicates were eliminated using Picard v2.9.0. GATK (v4.1.3.0) (https://github.com/broadinstitute/gatk/) was used for variant calling and filtering. Variant calling was conducted using GATK HaplotypeCaller. The variants from each sample were combined with GATK CombineGVCFs and genotyped using GATK GenotypeGVCFs. To ensure high‐quality variant calls, GATK VariantFiltration and SelectVariants were used for filtering, according to the following criteria: QD < 5.0, FS > 50.0, SOR > 3.0, MQ < 50.0, MQRankSum < −2.5, ReadPosRankSum < −1.0, and ReadPosRankSum > 3.5. Variant effects were annotated with rice genome annotations from the Rice Annotation Project Database (RAP‐DB; https://rapdb.dna.affrc.go.jp/) using SnpEff (Cingolani et al., 2012). Additionally, SnpSift v4.3t (Cingolani et al., 2012) was used to extract variants classified as having high or moderate impact effects.

### 2.5 Genetic map construction

Leaf tissue samples were collected from 3‐week‐old seedlings of each RIL and the two parental cultivars grown in the greenhouse. Genomic DNA was extracted using the DNeasy Plant Mini Kit (Qiagen). Genotyping was performed using a target capture sequencing SNP genotyping platform developed by C. Lee et al. (2022). A genetic map was constructed using MapDisto version 1.7.0 (Lorieux, 2012). The Kosambi mapping function was used to calculate the genetic distances between markers.

### 2.6 QTL analysis

A QTL mapping analysis was performed using the genetic map described above and the phenotypic trait data (PHS rates). Composite interval mapping was performed using the Windows QTL Cartographer version 2.5 (Basten et al., 2002) program. A permutation test with 1000 iterations was employed to determine the logarithm of odds (LOD) threshold at the significance level of 0.05. QTLs exceeding this threshold were considered significant. The genetic map and QTL positions were visualized using MapChart software (Voorrips, 2002).

### 2.7 Candidate gene identification

Sequence variation between Junam and Nampyeong within the QTL intervals was detected by comparing their genome sequences. Sequence variants with moderate or high impact effects on gene function were selected for further analysis. Variants within genes associated with seed germination and related biological pathways were prioritized and identified using RAP‐DB (http://rapdb.dna.affrc.go.jp) and a literature survey. Genes containing these variants were considered to be candidates for the PHS QTLs.

### 2.8 Development of derived cleaved amplified polymorphic sequence (dCAPS) marker

A dCAPS marker was developed based on the SNPs in *Os06g0317200*, the candidate gene for the QTL *qPHS6*. We used the dCAPS Finder 2.0 online tool (Neff et al., 2002) to design PCR primers and select a restriction enzyme that would distinguish between the Junam and Nampyeong alleles. PCR products were digested overnight with *Pvu*II at 37°C. The digested products were resolved by electrophoresis on a 3% agarose gel in 0.5x tris‐acetate‐EDTA buffer at 100 V for 2 h.

### 2.9 Genotyping using markers for *qLTG3‐1* and *Os06g0317200*

The RILs were genotyped using the InDel marker for *qLTG3‐1* and the dCAPS marker for *Os06g0317200*. The RIL genotypes were classified into four groups, AA, AB, BA, and BB, where “A” indicates the Junam allele and “B” the Nampyeong allele, while the first position represents the genotype at *qLTG3‐1* and the second position the genotype at *Os06g0317200*.

### 2.10 Haplotype analysis for *Os06g0317200*

Haplotype variation within *Os06g0317200* was analyzed using genotype data from the 3000 Rice Genomes Project database (https://snp‐seek.irri.org/download.zul). The SNPs located in the coding region of the gene were extracted. Variants in untranslated regions, introns, and synonymous sites were excluded.

### 2.11 Statistical analysis

A one‐way analysis of variance and Duncan's multiple range test were performed using SAS Enterprise Guide 8.3 (SAS Institute Inc.) to compare the mean PHS rates of each of the four genotype combinations (AA, AB, BA, and BB). Results were considered statistically significant at *p* < 0.05.

## 3 RESULTS

### 3.1 Development of a deep learning model for PHS measurement

**FIGURE 2:**
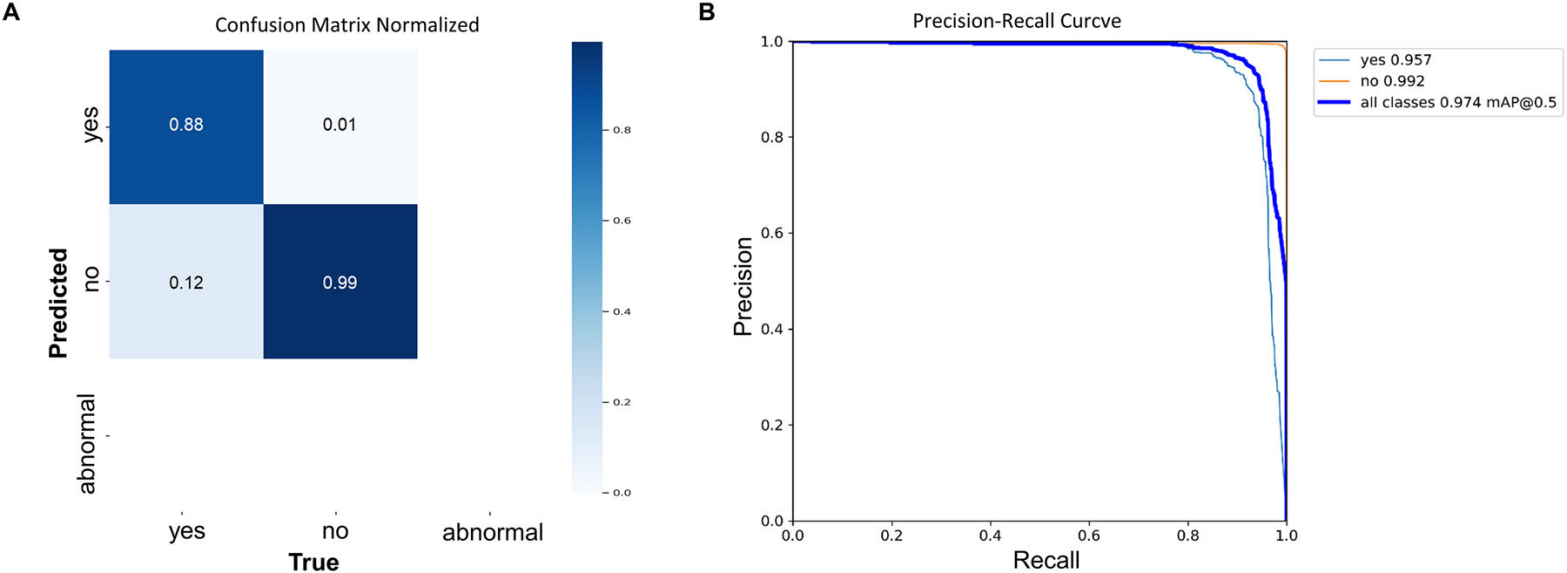
Performance evaluation of germination detection by the YOLOv8 model. (A) Confusion matrix describing the performance of the classification model by displaying the true and predicted labels. (B) Precision–recall (PR) curves for each class, which illustrate the model's precision.

Approximately 23,000 images of rice seeds after incubation for 5 days at 25°C to induce germination were produced. Of these, 18,000 images were used as training data and the remaining 5000 as validation data. Seeds were classified as “yes” (germinated), “no” (ungerminated), and “abnormal” (contaminated). The YOLOv8 model was trained over 229 epochs. Its performance was evaluated using a confusion matrix and precision–recall curves (Figure 2). The confusion matrix indicated that the classification performance of the model was higher for the “no” class (99%) than for the “yes” class (88%). Thus, the model identified ungerminated seeds with high accuracy, but was less accurate at identifying germinated seeds, with 12% of samples misclassified as “no.” The “abnormal” class contained insufficient labeled data, preventing meaningful evaluation of the model for this category (Figure 2A). The precision–recall curves highlight the model's performance for each class (Figure 2B). The model achieved a precision of 0.93, a recall of 0.909, and a mAP50 of 0.957 for the “yes” class, and a precision of 0.982, a recall of 0.997, and a mAP50 of 0.992 for the “no” class (Table 1).

**TABLE 1:** Assessment of the deep learning model for preharvest sprouting (PHS) rate measurement.

Class	Precision	Recall	mAP50	mAP50‐95
All	0.956	0.953	0.974	0.88
Yes	0.93	0.909	0.957	0.826
No	0.982	0.997	0.992	0.934

*Note*: Yes: germinated seeds. No: ungerminated seeds.

**FIGURE 3:**
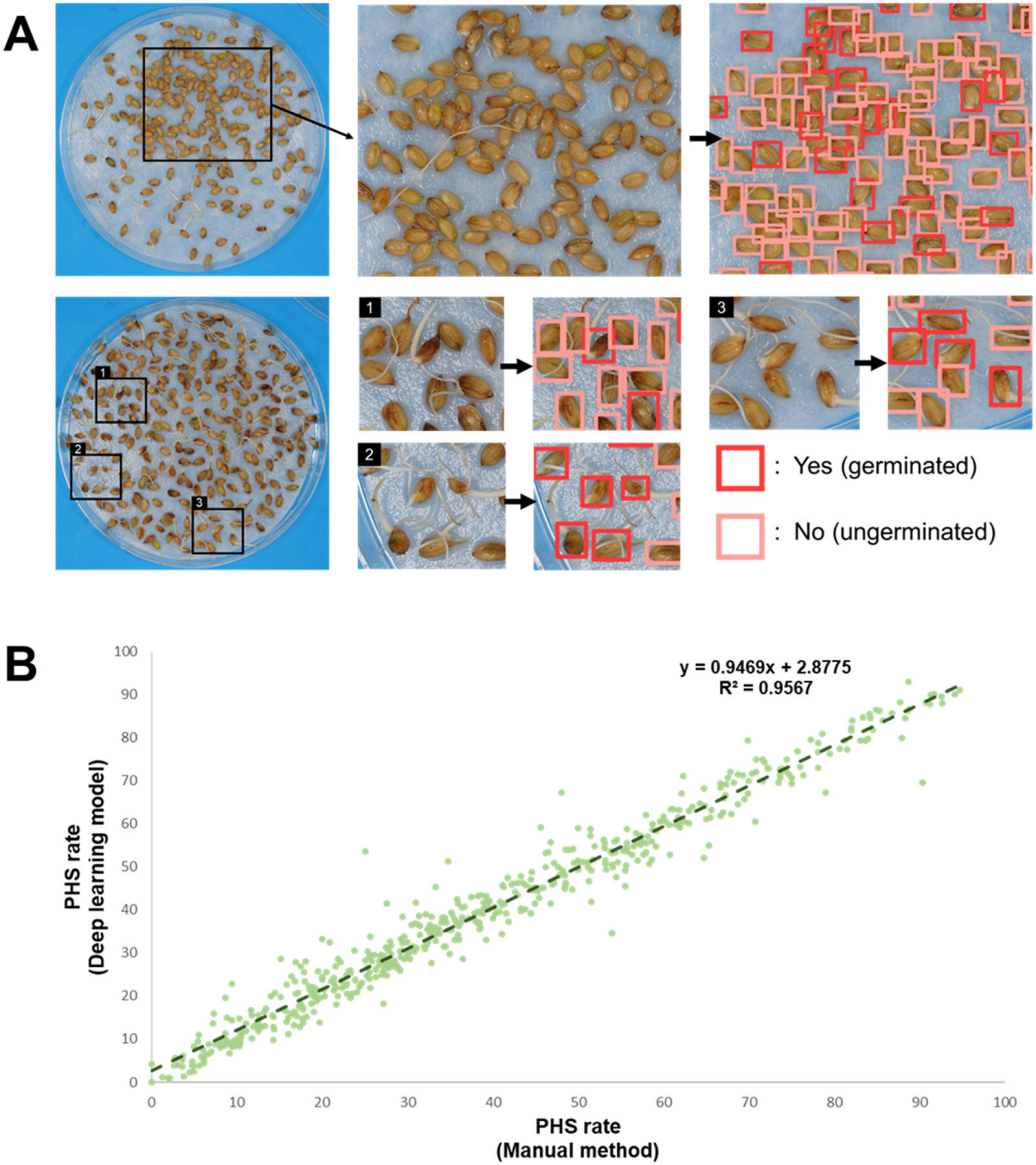
(A) Test of the deep learning model's ability to measure the seed germination rate. The model was capable of detecting seeds and discriminating between germinated and ungerminated seeds, even when the seeds were placed irregularly under high density conditions or partially obscured by sprouts. (B) Correlation between the preharvest sprouting (PHS) rates measured using the manual method and the deep learning model.

Seeds could be effectively detected and classified as “germinated” or “ungerminated”, even when they were arranged irregularly at a high density or if partially covered by sprouting radicles (Figure 3A). There was a scarcity of labeled data for the “abnormal” class, as contaminated seeds were a rare occurrence in this dataset, and thus the model's training for this class was less effective. Additionally, the absence of abnormal images in the validation set precluded the evaluation of this category. The trained model was applied to images of seeds from greenhouse‐grown plants to calculate their PHS rates. When the PHS rates from the deep learning model were compared with those from manual assessment, there was a very strong positive correlation (*R* ^2 ^= 0.9567) between the two methods (Figure 3B).

### 3.2 PHS rates in the RIL population

**FIGURE 4:**
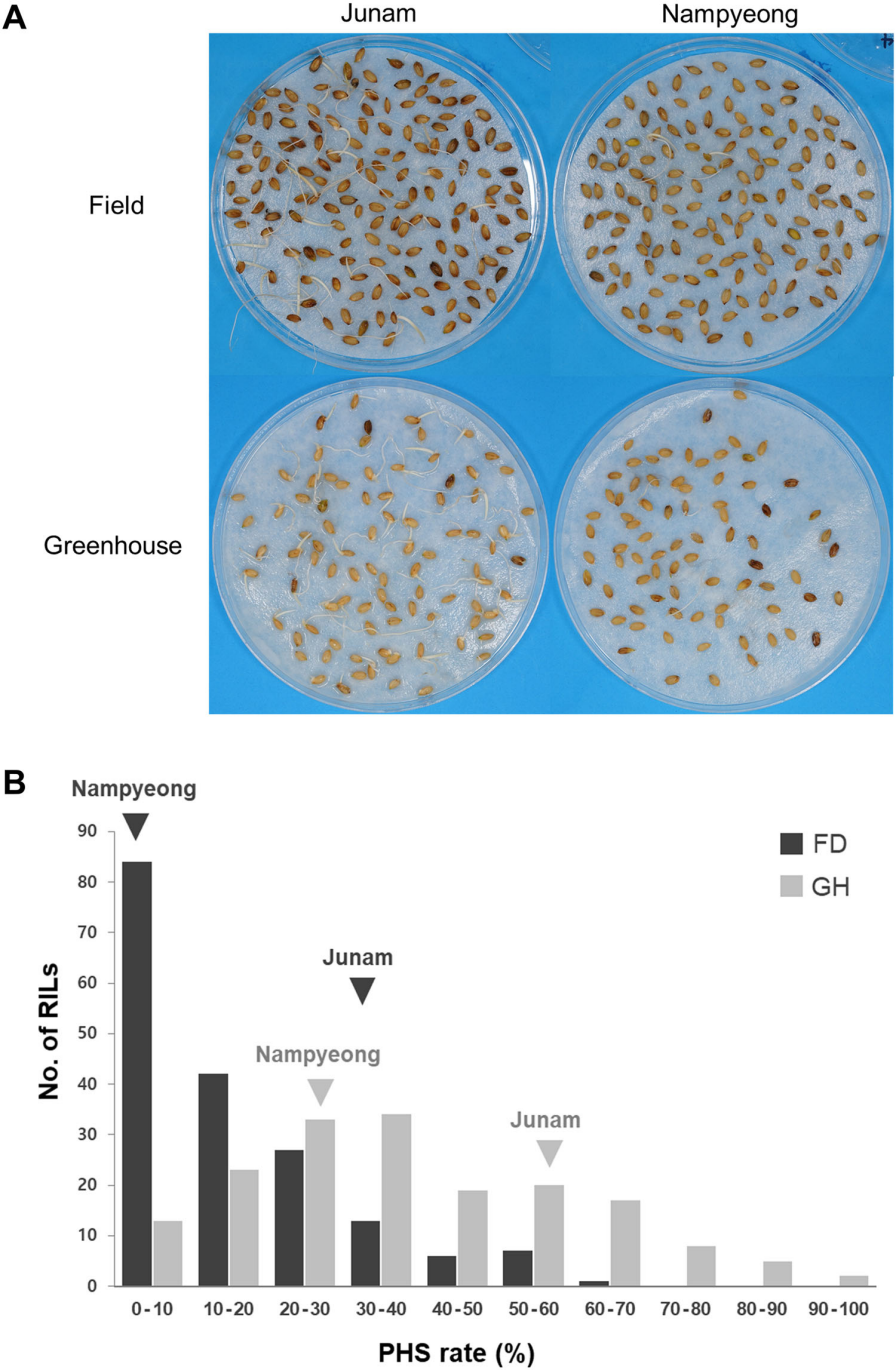
Distribution of preharvest sprouting (PHS) rates in recombinant inbred lines (RILs) derived from a cross between the *japonica* varieties Junam and Nampyeong grown under field and greenhouse conditions. (A) Representative images showing PHS in the parental varieties Junam and Nampyeong. (B) Distribution of PHS rates across RILs. Inverted triangles indicate the PHS rates of Junam and Nampyeong, black triangles indicate the field conditions, and gray triangles indicate the greenhouse conditions. FD, field conditions (black bars); GH, greenhouse conditions (gray bars).

Heading of the F_8_ RILs in the field was spread over 17 days from August 7, 2023. Heading of RILs grown in the greenhouse began on February 19, 2024, and took 36 days (Figure S2). The PHS rates ranged from 0.7% to 65.5% in the field, and from 3.0% to 93.1% in the greenhouse (Figure 4). The mean rate of PHS in the greenhouse was 38.6%, higher than the mean rate of 16.1% in the field. Junam had a mean PHS rate of 28.4% in the field and of 54.1% in the greenhouse; by contrast, the mean PHS rate of Nampyeong was 4.9% in the field and 26.9% in the greenhouse (Figure 4). QTL analyses were conducted on PHS rate data from 180 field‐grown RILs and 177 greenhouse‐grown RILs, as lines that died before harvest or had low seed counts were excluded from the dataset (Tables S1 and S2).

### 3.3 Sequence variation between parental varieties

The sequencing data from Junam and Nampyeong contained 424.65 × 10^6^ and 426.57 × 10^6^ raw reads, respectively. This corresponded to 55.20 Gbp of raw nucleotide data for Junam and 43.08 Gbp for Nampyeong. After quality filtering and mapping reads to the Nipponbare reference genome sequence (IRGSP‐1.0, https://rapdb.dna.affrc.go.jp/download/irgsp1.html), 385.75 × 10^6^ reads (49.67 Gbp) were mapped for Junam and 370.33 × 10^6^ reads (36.04 Gbp) for Nampyeong. The average mapped read depths were 111.83x for Junam and 78.90x for Nampyeong, ensuring high coverage and reliable variant detection (Table S3). Variant calling identified 445,564 polymorphic sites between the two varieties, made up of 371,830 SNPs and 73,734 InDels (Table S4).

### 3.4 Genetic map construction and QTL analysis

**FIGURE 5:**
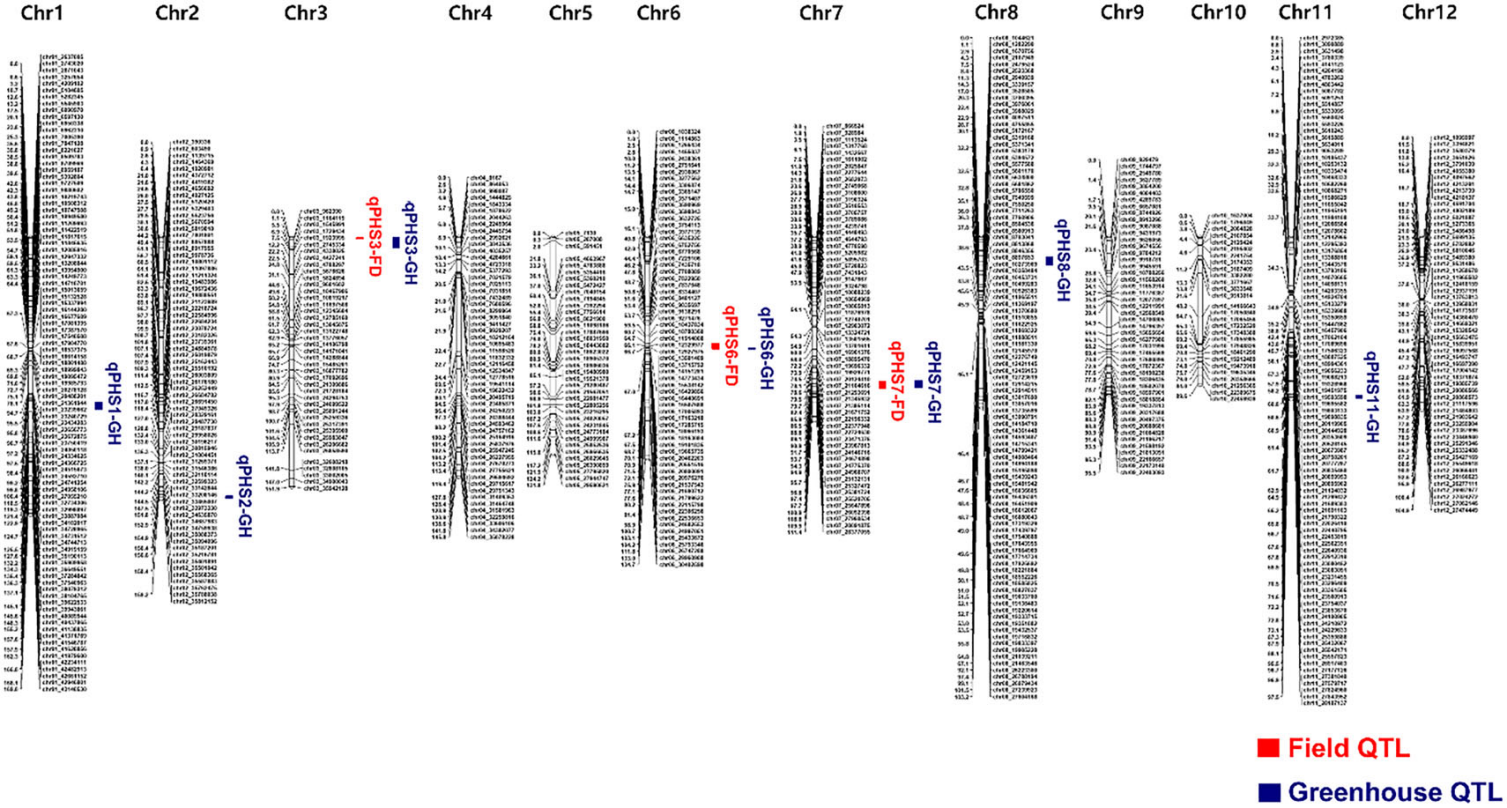
Genetic map of the Junam × Nampyeong F_8_ recombinant inbred line (RIL) population showing the locations of quantitative trait loci (QTLs) associated with preharvest sprouting (PHS) resistance under field (red) and greenhouse (blue) conditions.

A genetic map was constructed using 763 SNP markers identified through target‐capture sequencing of the 182 RILs (Table S5; Figure 5). The total map length was 1507.4 cM with an average marker interval of 2.0 cM (Table S6). QTL analyses were conducted by integrating the genotypic data from the SNP markers with the PHS rates of the RILs. Two QTL analyses were performed; one used phenotypic data from field‐grown RILs and the other used phenotypic data obtained under greenhouse conditions. An additive effect with a positive value indicated that the presence of the Junam allele increased the PHS rate, while a negative value indicated that the PHS rate decreased when the Junam allele was present.

Under field conditions, significant QTLs associated with PHS resistance were identified on chromosomes 3, 6, and 7 (Table 2; Figure 5). A major QTL located on chromosome 3 with an LOD score of 12.67 explained 20% of the variation in PHS rates (*R* ^2^ = 0.199). The position of this QTL corresponded with that of *qLTG3*‐*1*, a previously characterized gene that affects low temperature germination (Fujino et al., 2008) and PHS (Hori et al., 2010). A novel QTL on chromosome 6 had a LOD score of 4.45 (*R* ^2^ = 0.062). Another QTL with a LOD score of 4.67 (*R* ^2^ = 0.066) was identified on chromosome 7.

**TABLE 2:** Results of quantitative trait locus (QTL) mapping studies of preharvest sprouting (PHS) resistance under two environmental conditions.

Environment	QTL name	Chromosome	Position (cM)	LOD	Additive effect	*R* ^2^	QTL interval (cM)	Associated gene
Field	*qPHS3‐FD*	3	0.01	12.67	−6.27	0.199	0–1.0	*qLTG3‐1* (Fujino et al., 2008)
	*qPHS6‐FD*	6	66.21	4.45	3.49	0.062	64.5–68.1	
	*qPHS7‐FD*	7	89.21	4.67	3.69	0.066	87.6–91.9	
Greenhouse	*qPHS1‐GH*	1	101.81	10.34	8.50	0.157	100.5–104.9	
	*qPHS2‐GH*	2	158.51	3.36	−4.61	0.047	157.5–159.2	
	*qPHS3‐GH*	3	3.17	8.09	−7.31	0.119	0–6.5	*qLTG3‐1* (Fujino et al. 2008)
	*qPHS6‐GH*	6	67.61	10.87	8.65	0.161	67.1–68.1	
	*qPHS7‐GH*	7	89.51	3.65	4.50	0.045	87.2–91.8	
	*qPHS8‐GH*	8	14.41	7.65	6.99	0.107	11.9–16.6	
	*qPHS11‐GH*	11	97.69	3.99	−4.76	0.049	96.2–98.2	

*Note*: A positive additive effect indicates that the Junam allele increased the PHS rate; a negative additive effect indicates the Junam allele decreased the PHS rate.

Abbreviation: LOD, logarithm of odds.

Under greenhouse conditions, QTLs were detected on chromosomes 1, 2, 3, 6, 7, 8, and 11 (Table 2; Figure 5). The QTL *qPHS6* on chromosome 6 had the highest LOD score of 10.87 (*R* ^2^ = 0.161). Another significant QTL on chromosome 1 had a LOD score of 10.34 (*R* ^2^ = 0.157). The QTLs on chromosomes 2, 8, and 11 had LOD scores of 3.36 (*R* ^2^ = 0.047), 7.65 (*R* ^2^ = 0.107), and 3.99 (*R* ^2^ = 0.049), respectively. Notably, the QTLs on chromosomes 3, 6, and 7 were identified under both field and greenhouse environments, which demonstrated their consistent effect on PHS resistance. Furthermore, the results of the QTL mapping analysis using the PHS rates acquired by the deep learning model closely matched those of the QTL analysis that used the manual measurements of PHS rate (Figure S3), which verifies the usefulness of the deep learning model.

### 3.5 Analysis of a candidate gene for *qPHS6* and dCAPS marker development

**FIGURE 6:**
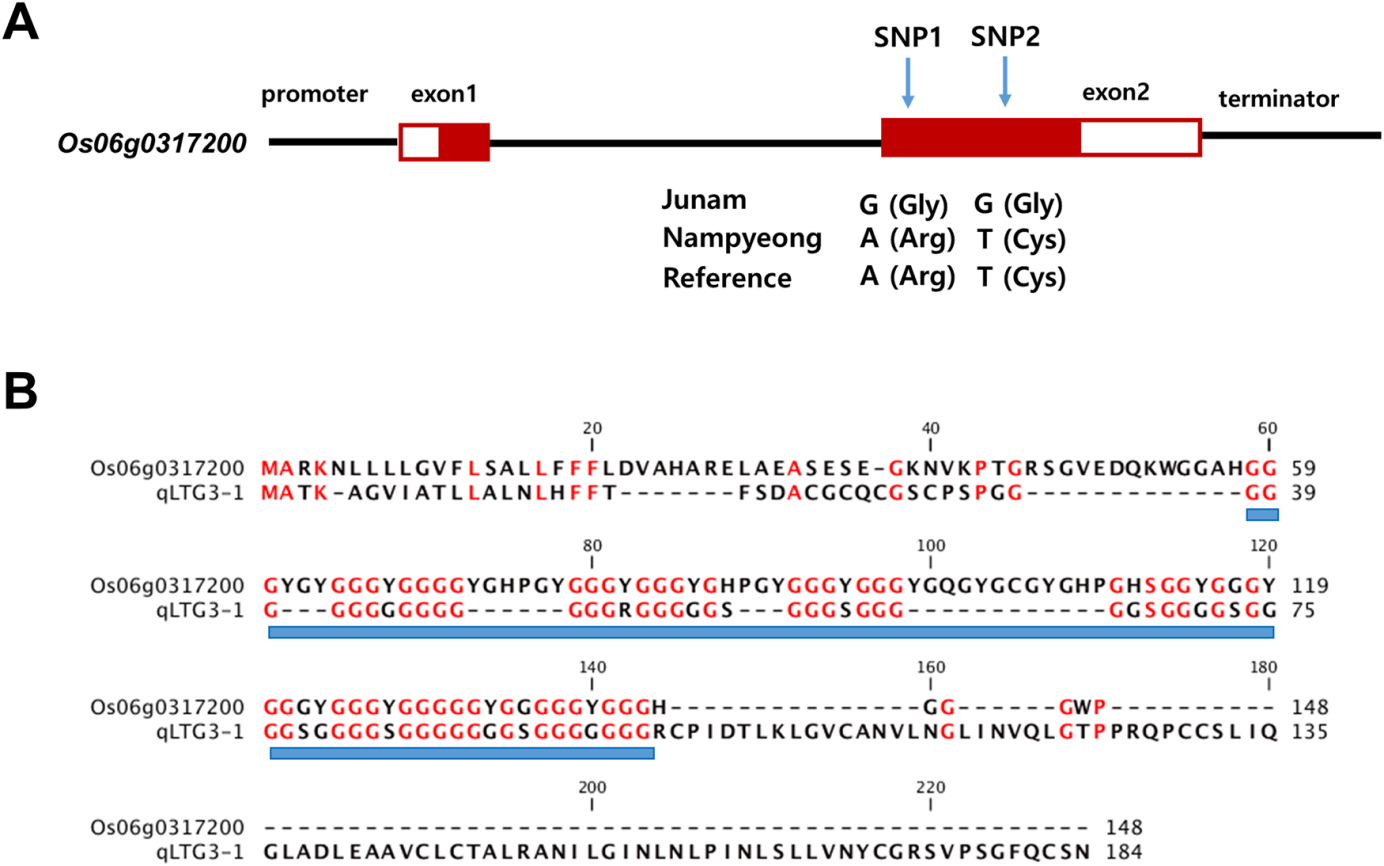
DNA sequence variations of *Os06g0317200* and protein amino acid sequence comparison between Os06g0317200 and qLTG3‐1. (A) Structure of *Os06g0317200* showing locations of single nucleotide polymorphisms (SNPs) that differ between Junam and Nampyeong. The reference sequence was the Nipponbare rice genome. Filled boxes, exons of coding sequence (CDS); empty boxes, 5′ and 3′ untranslated regions (UTRs). Black line between exons: intron. (B) Protein amino acid sequence alignment of Os06g0317200 and qLTG3‐1. Red‐colored residues indicate positions where the amino acid sequences are identical between the two proteins. Blue boxes indicate the glycine‐rich domain.

The QTL *qPHS6* exerted stable effects and had relatively high LOD scores in both field and greenhouse environments. We therefore performed an analysis to identify candidate genes at this locus. We identified genes within the *qPHS6* interval of 64.5–68.1 cM (10.78–19.10 Mbp) that contained SNPs with moderate or high impact effects on gene function (Table S7) and selected *Os06g0317200* as a potential candidate gene for *qPHS6*. This gene encodes a GRP similar to *qLTG3‐1*, which controls germinability at low temperature and PHS (Fujino et al. 2008; Hori et al., 2010). The second exon of *Os06g0317200* contained two SNPs with moderate impact effects that produced changes in the amino acid sequences between Junam and Nampyeong (Figure 6A). Nampyeong had an adenine nucleotide, which matched the reference genome, at the first SNP position while Junam had a guanine nucleotide at this position; this change resulted in the substitution of arginine with glycine. At the second SNP position, Nampyeong had a thymine nucleotide, which again matched the reference genome, whereas Junam had a guanine nucleotide at this position, causing the substitution of cysteine with glycine. Protein sequence alignment revealed a conserved glycine‐rich domain (Figure 6B) between Os06g0317200 and qLTG3‐1, suggesting a possible role of Os06g0317200 in dormancy regulation or seed germination. Moreover, the expression profile of *Os06g0317200* was very similar to that of *qLTG3‐1*, and *Os06g0317200* also showed high expression at the milk grain stage, which suggested it may play a role in seed development (Figure S4).

We developed a dCAPS marker against the first SNP within *Os06g0317200* (Table 3). Digestion of DNA from Junam resulted in a PCR band that was 24 bp shorter than that of Nampyeong, whose DNA remained undigested. This confirmed the polymorphism between the parental lines. In addition, we designed an InDel marker for *qLTG3‐1* based on the 71‐bp insertion/deletion (InDel) mutation (Hori et al., 2010) present in this gene (Table 3; Figure S5).

**TABLE 3:** Primer sequences of the derived cleaved amplified polymorphic sequence (dCAPS) marker for *Os06g0317200* and the InDel marker for qLTG3‐1.

Gene	Primer name	Sequence (5′–3′)	Restriction enzyme
*Os06g0317200*	*7200SNP1*_forward	AGGGGAAGAATGTGAAGCCAACAGCT	*Pvu*II
	*7200SNP1*_reverse	CACCATAGCCACCACCATAC	
*qLTG3‐1*	*qLTG3‐1*_forward	AAAGCTAGGTAGAGGCCAGG	–
	*qLTG3‐1*_reverse	GTTGATGGGGAGGTTGAGGT	

**FIGURE 7:**
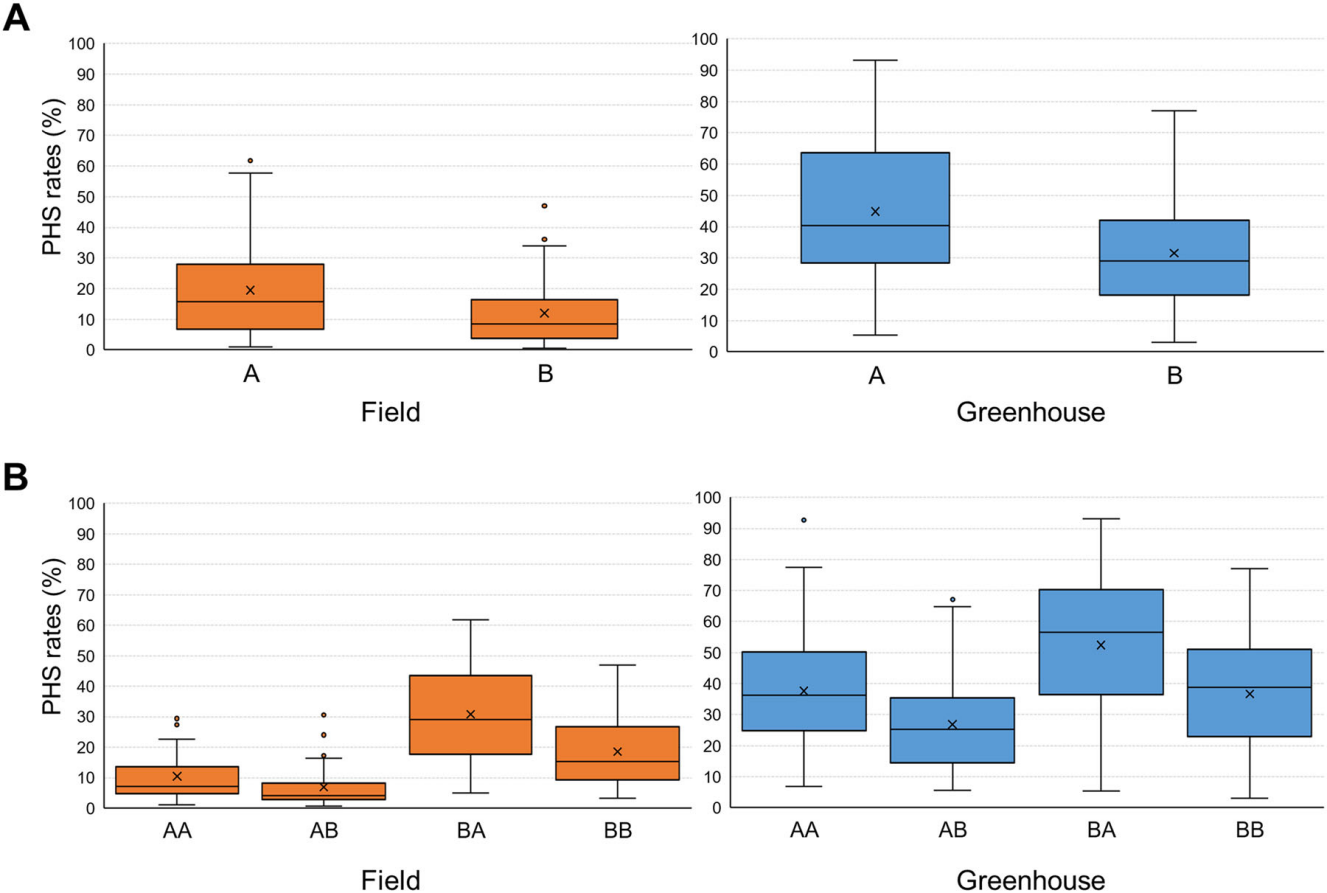
Boxplots showing preharvest sprouting (PHS) rates of recombinant inbred lines (RILs) derived from a cross between Junam and Nampyeong under field and greenhouse conditions. (A) PHS rates grouped by alleles at the *Os06g0317200* marker; A; Junam allele; B, Nampyeong allele. (B) PHS rates grouped by combined genotypes at two markers, *qLTG3‐1* and *Os06g0317200*. In each two‐letter genotype combination, first letter denotes genotype at *qLTG3‐1* and second letter denotes genotype at *Os06g0317200*.

The RILs were genotyped using both the *Os06g0317200* dCAPS and the *qLTG3‐1* InDel markers to determine the genotype segregation pattern (Figure S6) and the distribution of PHS rates across the different genotype combinations (Figure 7). As the population consisted of F_8_ RILs developed by repeated selfing, the genotypes had become nearly homozygous. Consequently, no heterozygous individuals were observed at either marker. To evaluate the independent effect of *Os06g0317200*, we first compared PHS rates between its two alleles. RILs carrying the Junam allele (A) showed higher mean PHS rates than those with the Nampyeong allele (B) in both field and the greenhouse experiments (Figure 7A). In addition, the PHS rates observed in the 182 RILs were grouped into the four possible genotype combinations (AA, AB, BA, and BB) at the markers *qLTG3‐1* and *Os06g0317200* (Figure 7B). RILs with the Junam allele (A) of *qLTG3‐1* had lower PHS rates whereas RILs with the Nampyeong allele (B) had higher PHS rates. Conversely, RILs with the Junam allele (A) of *Os06g0317200* had higher PHS rates and RILs with the Nampyeong allele (B) had lower PHS rates. RILs with the genotype BA had the highest mean PHS rate under both field (30.8%) and greenhouse (52.4%) conditions, whereas AB genotype RILs had the lowest mean rates of PHS (6.9% in the field and 26.8% in the greenhouse) (Figure 7B). The inverse relationship between the two markers highlighted their contrasting contributions to PHS regulation.

### 3.6 Patterns of haplotype variation in *Os06g0317200*

**FIGURE 8:**
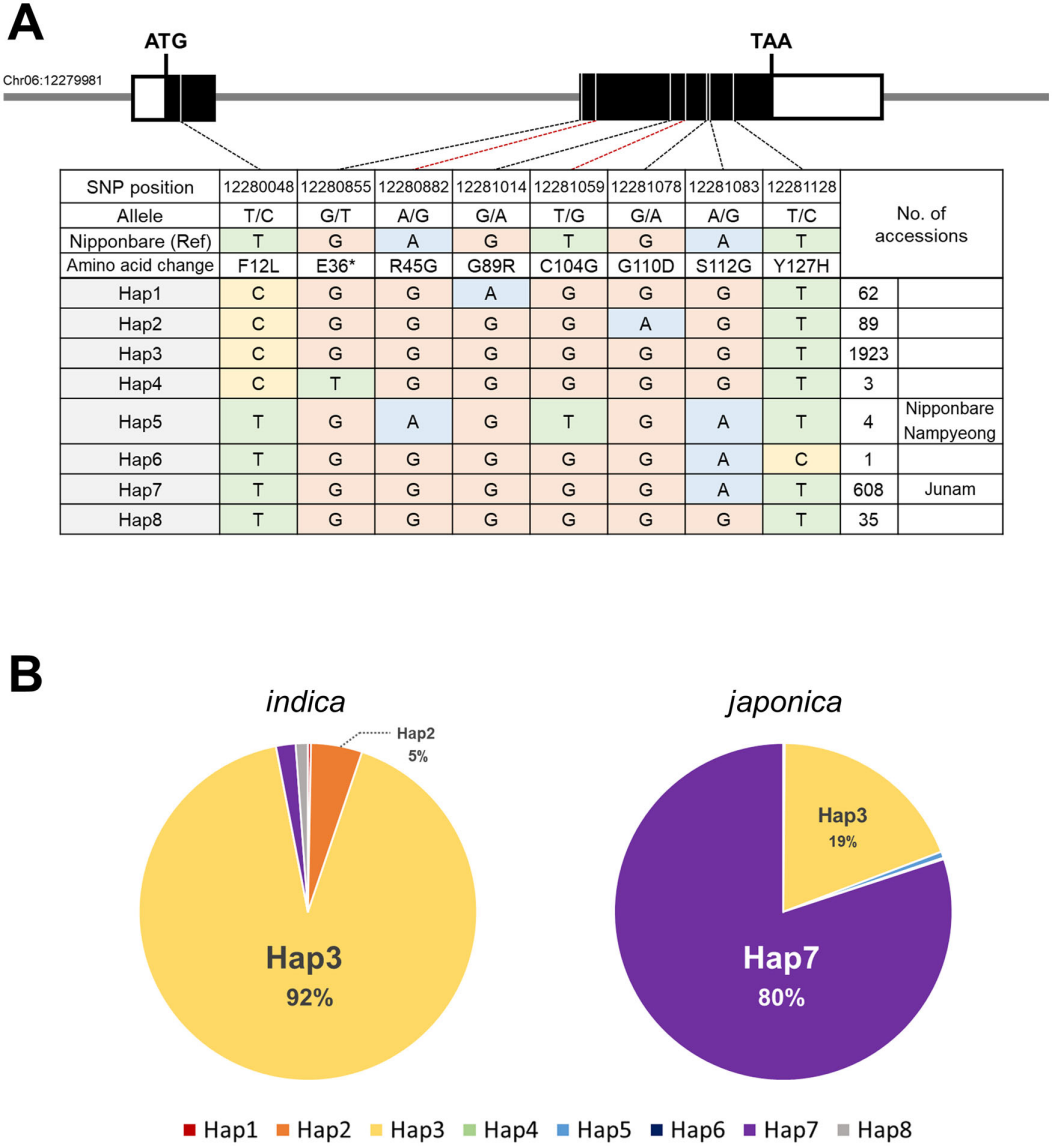
Haplotype structure of *Os06g0317200* and its distribution across rice subspecies. (A) Haplotype construction in *Os06g0317200* based on seven single nucleotide polymorphisms (SNPs) in the coding region. Exons are shown as two boxes, with untranslated regions (UTRs) represented by white regions and coding sequences (CDSs) by black regions. Red lines highlight the two SNPs between Junam and Nampyeong. (B) Distribution of haplotypes among *indica* and *japonica* accessions.

Allelic variation in *Os06g0317200* was analyzed across 2725 accessions from the 3000 Rice Genomes Project database (https://snp‐seek.irri.org/download.zul). Seven SNPs located in the coding region, including the two SNPs between Junam and Nampyeong, were selected to define haplotypes. This analysis resulted in the identification of eight distinct haplotypes (Figure 8A). Haplotypes exhibited clear subspecies‐specific patterns (Figure 8B). Hap3 predominated in *indica* (92%), while Hap7 was the most frequent in *japonica* (80%). Among the seven SNPs, one located in the first exon displayed a particularly sharp contrast between subspecies. The T allele was nearly fixed in *japonica*, whereas *indica* predominantly carried the C allele. This variant introduced a non‐synonymous amino acid change, implying potential functional divergence between the two groups.

Junam was classified as Hap7, the major haplotype among *japonica*. In contrast, both Nipponbare and Nampyeong harbored Hap5, which was rare and observed in only four accessions across the entire dataset. These results suggest that Junam represents a common haplotype in *japonica*, while Nipponbare and Nampyeong share an uncommon haplotype, highlighting genetic diversity within subspecies.

## 4 DISCUSSION

Accurate and efficient phenotyping is crucial for evaluating PHS and breeding PHS‐resistant cultivars (Cobb et al., 2013). Measuring the PHS rate manually is labor‐intensive and prone to human error, limiting the throughput of breeding programs (Sadeghi‐Tehran et al., 2019). To address this challenge, we developed a deep learning model using the YOLOv8 algorithm that automated the detection and scoring of germinated and ungerminated seeds. AI has been used previously to detect germination in seeds of various crops (Colmer et al., 2020; Genze et al., 2020; Q. Yao et al., 2024; J. Zhao et al., 2023), but little research has been dedicated toward developing an AI model specifically capable of detecting rice germination under high‐density conditions. We addressed this gap by testing up to 280 seeds in a 15 cm Petri dish. Our model maintained high accuracy, even when germinating sprouts obscured other seeds. We included images of field‐grown seeds in the training dataset to enhance our model's robustness when evaluating PHS rates. The model demonstrated high accuracy, detecting germinated seeds with a precision of 93%, recall of 90.9%, and mAP of 95.7%. This resulted in significant improvements in phenotyping efficiency. Despite its strengths, our deep learning model exhibited certain limitations; for instance, when the number of seeds exceeded 150, it occasionally assigned two bounding boxes to a single germinated seed. This issue could be resolved by adjusting the Intersection over Union threshold and increasing the confidence level during detection. In addition, our experiments were conducted using seeds placed on white filter paper and captured in an image‐acquisition box. It remains uncertain whether the model would retain its high accuracy under different background and lighting conditions. Further experiments are therefore required to evaluate its performance across diverse settings.

PHS occurs when grains germinate on the panicle before harvest and is typically triggered by adverse environmental factors, such as high humidity and temperature, during the maturation phase (Mares & Mrva, 2014). It is a significant agronomic issue in rice cultivation that results in substantial yield losses and a reduction in grain quality worldwide (Shu et al., 2015). Understanding the genetic and environmental factors influencing PHS is crucial for developing PHS‐resistant cultivars and ensuring food security in the context of ongoing climate change (Patwa & Penning, 2023). The PHS rate of the RIL population used in this study was significantly higher under greenhouse conditions (mean PHS rate: 38.6%) than in the field (mean PHS rate: 16.1%). This suggested that the greenhouse environment, which was characterized by a relatively high constant temperature, may have created conditions favorable for PHS (Figure S1). High temperature during grain filling leads to low amylose levels in rice endosperm due to a decrease in Wx/GBSSI activity, as well as a low starch crystallinity with more pitting and starch granules; all these are potential factors for increasing PHS (H. Lee, Choi, et al., 2021; D. Yao et al., 2020). Rising temperatures and altered rainfall patterns caused by climate change are likely to increase the future risk of PHS (Patwa & Penning, 2023). The LOD score of *qPHS6* under greenhouse conditions (LOD = 10.87) was higher than in the field (LOD = 4.45), suggesting a stronger genetic effect in an environment with relatively high temperature during seed maturation. This indicates the importance of this locus on PHS resistance in an environment that mimics future climatic scenarios.

*Os06g0317200*, the candidate gene for *qPHS6*, encoded a GRP. GRP is a family of diverse proteins that are characterized by glycine‐rich motifs, which contribute to their structural flexibility and ability to interact with a variety of cellular components (Czolpinska & Rurek, 2018; Sachetto‐Martins et al., 2000). GRPs are involved in multiple plant processes, including development, stress responses, RNA binding, and cell wall formation (Mangeon et al., 2010; Ringli et al., 2001). *Os06g0317200* resembles *qLTG3‐1*, a gene that affects PHS and seed germination under low temperature conditions (Fujino et al., 2008; Hori et al., 2010). *qLTG3‐1* encodes a GRP that promotes germination, probably by weakening the tissue surrounding the embryo and promoting water uptake (Fujino et al., 2008). In our study, the PHS‐resistant cultivar Nampyeong had a functional *qLTG3‐1* allele, whereas the intermediate‐susceptible cultivar Junam had a 71 bp deletion in *qLTG3‐1*, resulting in a loss‐of‐function mutation (Hori et al., 2010). In Junam, two SNPs in the *Os06g0317200* sequence resulted in amino acid substitutions that would potentially alter the protein's function. The Junam allele may thus increase the PHS rate under conditions of high temperature and humidity, whereas the Nampyeong allele may contribute to PHS resistance. Moreover, GRPs are characterized by their high glycine content, which contributes to protein structure by enabling the formation of random coils or β‐pleated sheets (Campini et al., 2022; Ringli et al., 2001). Amino acid substitutions that introduce additional glycine residues, as in the Junam sequence, may alter the structural dynamics of the protein and affect its flexibility. Such changes in GRP structure could also affect its interactions with other cell wall components and so alter the mechanical properties of the seed coat (Ringli et al., 2001).

Haplotype analysis of *Os06g0317200* revealed clear subspecies‐specific patterns, with Hap3 dominating in *indica* and Hap7 being most frequent in *japonica*. Among the eight defined haplotypes, Hap5—carried by Nampyeong and Nipponbare—was extremely rare, accounting for only four out of 2725 accessions in the 3K panel. Interestingly, the two SNPs that distinguish Nampyeong and Nipponbare from Junam were not only divergent between these varieties, but also uncommon within the *japonica* subgroup. This suggests that Nampyeong and Nipponbare harbor a rare allelic variant of *Os06g0317200* that is not widely represented in current *japonica* germplasm. Although phenotypic data for PHS are not available for the 3K accessions, the presence of this rare haplotype in PHS‐resistant varieties raises the possibility that allelic diversity at *Os06g0317200* may contribute to variation in dormancy or germination behavior. Further functional studies will be needed to determine the potential role of this gene in regulating PHS‐related traits.

## 5 CONCLUSION

We identified a major QTL for PHS resistance (*qPHS6*) that explained a high proportion of the variation in PHS rates in both field and greenhouse environments in an F_8_ RIL population derived from a cross between *japonica* rice varieties Junam and Nampyeong. We also developed a deep learning model for measuring the PHS rate that can be used to produce phenotype data for QTL and association mapping studies. These results will enable the acceleration of breeding programs to produce PHS‐resistant rice varieties.

## AUTHOR CONTRIBUTIONS

**Soojin Jun**: Data curation; investigation; methodology; software; writing—original draft. **Mi Hyun Cho**: Investigation. **Hyoja Oh**: Investigation. **Younguk Kim**: Methodology; software; visualization. **Dong Kyung Yoon**: Investigation. **Myeongjin Kang**: Investigation. **Hwayoung Kim**: Investigation. **Seon‐Hwa Bae**: Investigation. **Song Lim Kim**: Resources. **Jeongho Baek**: Methodology; software. **HwangWeon Jeong**: Methodology; software. **Jae Il Lyu**: Resources. **Gang‐Seob Lee**: Resources. **Changsoo Kim**: Conceptualization; writing—review and editing. **Hyeonso Ji**: Conceptualization; funding acquisition; investigation; project administration; supervision; writing—review and editing.

## CONFLICT OF INTEREST STATEMENT

The authors declare no conflicts of interest.

## Supporting information



Supplementary Material

Supplementary Material

Supplementary Material

Supplementary Material

## Data Availability

All data generated or analyzed during this study are included in this published article. The genome sequencing data of Junam and Nampyeong are available in the Sequence Read Archive (SRA) database of NCBI (https://www.ncbi.nlm.nih.gov/sra) under the accession number PRJNA528640 and PRJNA1256927, respectively.
