# Supplementary material for: Mapping QTLs for PHS resistance and development of a deep learning model to measure PHS rate in japonica rice
Source: Plant Genome. 2025 Aug 30;18(3):e70109. doi: 10.1002/tpg2.70109 (PMC12397900; doi:10.1002/tpg2.70109)
Supplement: Supplementary file 1 — Supplementary Material [file TPG2-18-e70109-s004.docx]

Table S3. Summary of genomic sequencing data from the parental *japonica* rice varieties Junam and Nampyeong

| **Variety** | **Raw Sequencing Data** | |  | **After Quality Trimming (Q20*)** | | |  | **After Read Mapping** | | |
| --- | --- | --- | --- | --- | --- | --- | --- | --- | --- | --- |
|  | **No. of Reads**  **(×10^6^)** | **Nucleotides**  **(Gbp **)** |  | **No. of Reads**  **(×10^6^)** | **Nucleotides (Gbp)** | **Sequencing Depth (×)** |  | **No. of reads (×10^6^)** | **Nucleotides**  **(Gbp)** | **Average**  **Mapping**  **Depth (×)** |
| Junam | 424.65 | 55.2 |  | 385.75 | 49.67 | 133.09 |  | 322.89 | 41.74 | 111.83 |
| Nampyeong | 426.57 | 43.08 |  | 370.33 | 36.04 | 96.6 |  | 302.28 | 29.45 | 78.9 |

* The Q20 value indicates an accuracy of 99% for the base called

** Gbp: 10^9^ bp.

Table S4. Numbers of sequence polymorphisms (SNPs and InDels) between Junam and Nampyeong

| **Chr.** | **SNP** | **InDel** | **Total** |
| --- | --- | --- | --- |
| 1 | 29,344 | 6,866 | 36,210 |
| 2 | 7,563 | 2,425 | 9,988 |
| 3 | 13,711 | 3,436 | 17,147 |
| 4 | 14,346 | 3,178 | 17,524 |
| 5 | 1,056 | 585 | 1,641 |
| 6 | 53,601 | 8,145 | 61,746 |
| 7 | 19,947 | 5,026 | 24,973 |
| 8 | 87,850 | 15,139 | 102,989 |
| 9 | 10,517 | 2,433 | 12,950 |
| 10 | 11,085 | 2,412 | 13,497 |
| 11 | 103,990 | 19,306 | 123,296 |
| 12 | 1,8820 | 4,783 | 23,603 |
| Total | 371830 | 73,734 | 445,564 |

Table S6. Distribution of SNP markers across a genetic map constructed through target capture sequencing of the F_8_ RIL population derived from a cross between Junam and Nampyeong

| **Chr.** | **No. of**  **SNP markers** | **Map distance**  **(cM)** | **Marker density (SNP/cM)** |
| --- | --- | --- | --- |
| 1 | 96 | 170.5 | 0.6 |
| 2 | 70 | 160.1 | 0.4 |
| 3 | 42 | 153.5 | 0.3 |
| 4 | 55 | 147.5 | 0.4 |
| 5 | 38 | 133.5 | 0.3 |
| 6 | 66 | 135.6 | 0.5 |
| 7 | 62 | 112.3 | 0.6 |
| 8 | 100 | 103.7 | 1.0 |
| 9 | 48 | 96.3 | 0.5 |
| 10 | 28 | 90.4 | 0.3 |
| 11 | 101 | 98.2 | 1.0 |
| 12 | 57 | 105.9 | 0.5 |
| Total | 763 | 1507.4 |  |
| Mean | 63.6 | 125.6 | 0.5 |

Table S7. List of genes in the *qPHS6* region containing sequence differences between the Junam and Nampyeong genomes that were predicted to have moderate or high impact effects on gene function

| **Gene ID** | **Gene description** | **No. of**  **HIEV** | **No. of**  **MIEV** |
| --- | --- | --- | --- |
| Os06g0317200 | Similar to glycine-rich cell wall structural protein. |  | 2 |
| Os06g0328900 | Cytochrome P450 family protein. |  | 1 |
| Os06g0329900 | SAM dependent carboxyl methyltransferase family protein. |  | 1 |
| Os06g0330100 | Blast resistance protein, Pseudogene due to premature stop mutation (most japonica varieties), Blast resistance | 1 | 5 |
| Os06g0330400 | Fatty acid hydroxylase domain containing protein. | 1 | 7 |
| Os06g0331900 | Uncharacterized protein family UPF0005 domain containing protein. |  | 1 |
| Os06g0332400 | VHS domain containing protein. |  | 3 |
| Os06g0332600 | Similar to Rf2 protein. | 1 | 1 |
| Os06g0332900 | Similar to Zn-finger, RanBP-type, containing protein. |  | 3 |
| Os06g0334400 | Similar to Cdk-activating kinase 1At (Cdk-activating kinase CAK1At). |  | 1 |
| Os06g0335101 | Micro-fibrillar-associated 1, C-terminal domain containing protein. |  | 1 |
| Os06g0335500 | AUX/IAA protein family protein. |  | 2 |
| Os06g0335950 | Non-protein coding transcript. | 2 |  |
| Os06g0335900 | Concanavalin A-like lectin/glucanase domain containing protein. | 2 | 5 |
| Os06g0336200 | Delta-tonoplast intrinsic protein. |  | 1 |
| Os06g0336500 | Cellulose synthase like protein D5 |  | 1 |
| Os06g0337200 | Similar to ZF-HD homeobox protein. |  | 4 |
| Os06g0338200 | Copper amine oxidase family protein. |  | 3 |
| Os06g0338700 | Similar to Primary amine oxidase. | 1 | 12 |
| Os06g0338900 | Bacterial transferase hexapeptide repeat domain containing protein. |  | 1 |
| Os06g0339800 | Ribosomal protein L30, bacterial family protein. |  | 1 |
| Os06g0340200 | Zinc finger, RING-CH-type domain containing protein. |  | 1 |
| Os06g0340600 | Similar to phosphatidylinositol 3- and 4-kinase family protein. |  | 3 |
| Os06g0341300 | Seed maturation protein domain containing protein. |  | 2 |
| Os06g0341801 | Similar to Maturase K (Fragment). |  | 1 |
| Os06g0342000 | Exostosin-like family protein. |  | 2 |
| Os06g0342500 | K Homology, type 1, subgroup domain containing protein. |  | 1 |
| Os06g0342750 | Similar to predicted protein. | 1 | 1 |
| Os06g0343100 | Similar to ATP-dependent helicase DHX8 (RNA helicase HRH1) (DEAH-box protein 8). |  | 10 |
| Os06g0343900 | Armadillo-like helical domain containing protein. |  | 2 |
| Os06g0344900 | NAC transcription factor |  | 1 |
| Os06g0345200 | Similar to Nicotianamine aminotransferase. |  | 3 |
| Os06g0346300 | Similar to ACX4 (ACYL-COA OXIDASE 4); acyl-CoA oxidase/ oxidoreductase. | 1 | 3 |
| Os06g0347100 | Similar to Acyl-coenzyme A oxidase 4, peroxisomal (EC 1.3.3.6) (AOX 4) (Short- chain acyl-CoA oxidase) (SAOX) (AtCX4) (G6p) (AtG6). |  | 1 |
| Os06g0348800 | GARP transcription factor, Orthologous to maize Golden2-like 1, Regulation of chloroplast development |  | 3 |
| Os06g0349700 | Ferulate-5-hydroxylase, Cytochrome P450-dependent monooxygenase | 1 | 4 |
| Os06g0350600 | Similar to Pathogenesis-related protein 1 (Fragment). | 1 | 5 |
| Os06g0351500 | Lipase, GDSL domain containing protein. |  | 1 |
| Os06g0352200 | Protein of unknown function DUF679 family protein. |  | 2 |
| Os06g0352900 | Photosystem II (PSII) auxiliary protein, Regulation of D1 protein stability of PSII |  | 4 |
| Os06g0353400 | Similar to Histone mRNA exonuclease 1. |  | 3 |
| Os06g0354500 | Acyl-CoA oxidase 3 |  | 2 |
| Os06g0354700 | Alpha/beta hydrolase-fold family protein, Chlorophyll degradation during senescence |  | 2 |
| Os06g0355500 | Bromodomain containing protein. |  | 1 |
| Os06g0356800 | Similar to Xylanase inhibitor protein I precursor. |  | 1 |
| Os06g0357000 | Protein of unknown function DUF1409 domain containing protein. |  | 5 |
| Os06g0358800 | Ribonuclease III domain containing protein. | 2 | 2 |
| Os06g0359400 | Ribosomal protein L18a domain containing protein. | 1 | 1 |
| Os06g0360300 | NAD(P)-binding domain containing protein. |  | 1 |
| Os06g0360600 | Similar to OSIGBa0104J13.3 protein. |  | 2 |
| Os06g0367100 | Glycoside hydrolase, subgroup, catalytic core domain containing protein. |  | 4 |
| Os06g0367500 | Similar to B-cell receptor-associated protein 31-like containing protein. |  | 3 |
| Os06g0367900 | Similar to Mitogen-activated protein kinase homologue. |  | 5 |
| Os06g0469800 | Similar to H0124E07.4 protein. |  | 3 |
| Os06g0470000 | Glycosyltransferase AER61, uncharacterized domain containing protein. |  | 2 |
| Os06g0470800 | Protein of unknown function DUF159 family protein. |  | 4 |
| Os06g0472000 | Methionine sulfoxide reductase B1, Oxidative stress tolerance, Salt tolerance |  | 1 |
| Os06g0472200 | Similar to Isoflavone reductase. |  | 4 |
| Os06g0472300 | Pentatricopeptide repeat domain containing protein. |  | 1 |
| Os06g0472400 | Similar to Transcriptional regulator. |  | 2 |
| Os06g0472900 | Heme peroxidase family protein. |  | 1 |
| Os06g0473000 | Poor homologous synapsis 1 (PHS1) protein, Male and female gamete development |  | 1 |
| Os06g0473100 | Similar to signal recognition particle receptor beta subunit. |  | 2 |
| Os06g0473200 | Similar to NPK2. |  | 2 |
| Os06g0474200 | Subunit of RNA N6-methyladenosine methyltransferase, Regulation of sporogenesis and embryo development, Male gametogenesis |  | 2 |
| Os06g0474300 | Methyltransferase type 11 domain containing protein. |  | 1 |
| Os06g0474500 | Protein of unknown function DUF239, plant domain containing protein. |  | 2 |
| Os06g0474800 | Similar to 3Fe-4S ferredoxin. |  | 1 |
| Os06g0474866 | Similar to Alpha-glucan water dikinase (Fragment). |  | 1 |
| Os06g0475400 | Glycosyltransferase AER61, uncharacterized domain containing protein. |  | 2 |
| Os06g0476100 | Similar to GRAS family transcription factor containing protein, expressed. |  | 1 |
| Os06g0476200 | Similar to Phosphoglucomutase precursor (EC 5.4.2.2). |  | 4 |
| Os06g0478600 | Leucine-rich repeat, N-terminal domain containing protein. |  | 2 |
| Os06g0479400 | Similar to Phenylcoumaran benzylic ether reductase homolog TH6. | 1 | 2 |
| Os06g0480000 | Zinc finger, RING/FYVE/PHD-type domain containing protein. |  | 4 |
| Os06g0483100 | Similar to Luminal-binding protein 4. |  | 1 |
| Os06g0483150 | Glutamate dehydrogenase, NAD-specific domain containing protein. | 1 |  |
| Os06g0483200 | Similar to cycloartenol synthase. | 1 | 3 |
| Os06g0483500 | Similar to H0124B04.15 protein. | 2 | 3 |
| Os06g0483900 | Homeodomain-like containing protein. |  | 7 |
| Os06g0484450 | Similar to Chlorophyll a-b binding protein 2, chloroplastic. | 3 | 20 |
| Os06g0484600 | Similar to Pherophorin-S precursor. |  | 2 |
| Os06g0484800 | Reverse transcriptase domain containing protein. |  | 5 |
| Os06g0485100 | Similar to Homeobox-like resistance. |  | 3 |
| Os06g0486000 | Proline-rich extensin-like receptor kinase 6, Response to jasmonic acid (JA), abscisic acid (ABA) and brassinosteroid treatment, Response to M. oryzae infection |  | 5 |
| Os06g0486300 | Similar to MLO protein homolog 1. |  | 2 |
| Os06g0486400 | Serine/threonine protein kinase domain containing protein. |  | 1 |
| Os06g0486800 | Similar to Formate dehydrogenase, mitochondrial precursor (EC 1.2.1.2) (NAD- dependent formate dehydrogenase) (FDH). |  | 1 |
| Os06g0486900 | Similar to Formate dehydrogenase, mitochondrial precursor (EC 1.2.1.2) (NAD- dependent formate dehydrogenase) (FDH). |  | 1 |
| Os06g0487900 | SUMO (Small Ubiquitin-like Modifier) Protease, Salt tolerance |  | 2 |
| Os06g0488050 | Similar to Protein kinase family protein. |  | 1 |
| Os06g0489200 | Protein of unknown function DUF1604 domain containing protein. |  | 5 |
| Os06g0489500 | CMP/dCMP deaminase, zinc-binding domain containing protein. |  | 16 |
| Os06g0489900 | Domain of unknown function DUF1618 domain containing protein. | 1 | 2 |
| Os06g0490000 | Similar to DIMETHYLADENOSINE TRANSFERASE. |  | 2 |
| Os06g0490400 | Similar to Class III peroxidase 80. |  | 1 |
| Os06g0491800 | HAT dimerization domain containing protein. | 1 | 2 |
| Os06g0492000 | Similar to Phosphatidylinositol synthase. | 2 | 1 |
| Os06g0492700 | Similar to Dynein light chain LC6, flagellar outer arm. |  | 1 |
| Os06g0492900 | F-box domain, cyclin-like domain containing protein. |  | 3 |
| Os06g0493100 | Insect-inducible protein homologous to wheat Wir1, Resistance to the brown planthopper (BPH) |  | 2 |
| Os06g0494100 | S-Domain receptor like kinase-PID2, Receptor-like serine-threonine kinase protein, B-lectin receptor kinase, Blast disease resistance, Resistance to M. grisea strain ZB15 (Nipponbare: susceptible) |  | 2 |
| Os06g0494400 | Multi antimicrobial extrusion protein MatE family protein. |  | 5 |
| Os06g0495100 | Similar to cDNA, clone: J100088H20, full insert sequence. |  | 1 |
| Os06g0495632 | Similar to F-box domain containing protein. |  | 4 |
| Os06g0495500 | Multi antimicrobial extrusion protein MatE family protein. |  | 1 |
| Os06g0495700 | Beta tubulin, autoregulation binding site domain containing protein. |  | 3 |
| Os06g0495800 | Protein of unknown function DUF617, plant family protein. | 1 |  |
| Os06g0496000 | Like-Sm ribonucleoprotein (LSM)-related domain domain containing protein. |  | 6 |
| Os06g0496400 | ROOT HAIR DEFECTIVE-SIX LIKE (RSL) class I basic helix-loop-helix protein, bHLH transcription factor, Regulation of root hair development |  | 3 |
| Os06g0496800 | Membrane-associated kinase-6, Response to submergence |  | 2 |
| Os06g0497350 | Similar to Cytochrome P450 CYP71K14. |  | 2 |
| Os06g0497600 | Similar to H0321H01.8 protein. |  | 1 |
| Os06g0498150 | Regulation of seed development |  | 1 |
| Os06g0498400 | Alpha-glucan, water dikinase, Positive regulation of seed germination, Tolerance to abiotic stress, Regulation of grain yield and quality |  | 5 |
| Os06g0498500 | CCAAT-binding factor domain containing protein. |  | 2 |
| Os06g0499301 | Pentatricopeptide repeat domain containing protein. |  | 10 |
| Os06g0499500 | Similar to Indole-3-acetic acid-amido synthetase GH3.17 (EC 6.3.2.-) (Auxin- responsive GH3-like protein 17) (AtGH3-17). |  | 3 |
| Os06g0500700 | Cytochrome P450 family protein. |  | 9 |
| Os06g0501900 | Cytochrome P450 family protein. |  | 1 |
| Os06g0502800 | Similar to Plant protein family protein, expressed. |  | 4 |
| Os06g0503400 | Reticulon family protein. |  | 1 |
| Os06g0503850 | Similar to Acyl-[acyl-carrier-protein] desaturase 7, chloroplastic. | 2 | 1 |
| Os06g0504100 | Similar to SRF-type transcription factor family protein, expressed. |  | 1 |
| Os06g0504900 | WRKY transcription factor 31, Bacterial leaf blight resistance |  | 5 |
| Os06g0505302 | Similar to SWEETIE (SWEETIE); binding. |  | 4 |
| Os06g0505400 | Abortive infection protein family protein. |  | 1 |
| Os06g0505700 | Similar to AGP16. |  | 1 |
| Os06g0506100 | Pentatricopeptide repeat domain containing protein. |  | 2 |
| Os06g0507100 | Similar to Prolamin. | 1 | 1 |
| Os06g0507200 | Bifunctional inhibitor/plant lipid transfer protein/seed storage domain containing protein. |  | 1 |
| Os06g0507400 | Similar to GAMYB-binding protein (Fragment). |  | 1 |
| Os06g0509100 | Serine/threonine protein kinase-related domain containing protein. | 1 |  |
| Os06g0509600 | Glycoside hydrolase, family 28 domain containing protein. |  | 1 |
| Os06g0509900 | Similar to Thionin. |  | 1 |
| Os06g0520600 | Similar to Zinc finger CCCH type domain containing protein ZFN-like 1. | 1 |  |

* HIEV: high impact effect variation, MIEV: moderate impact effect variation


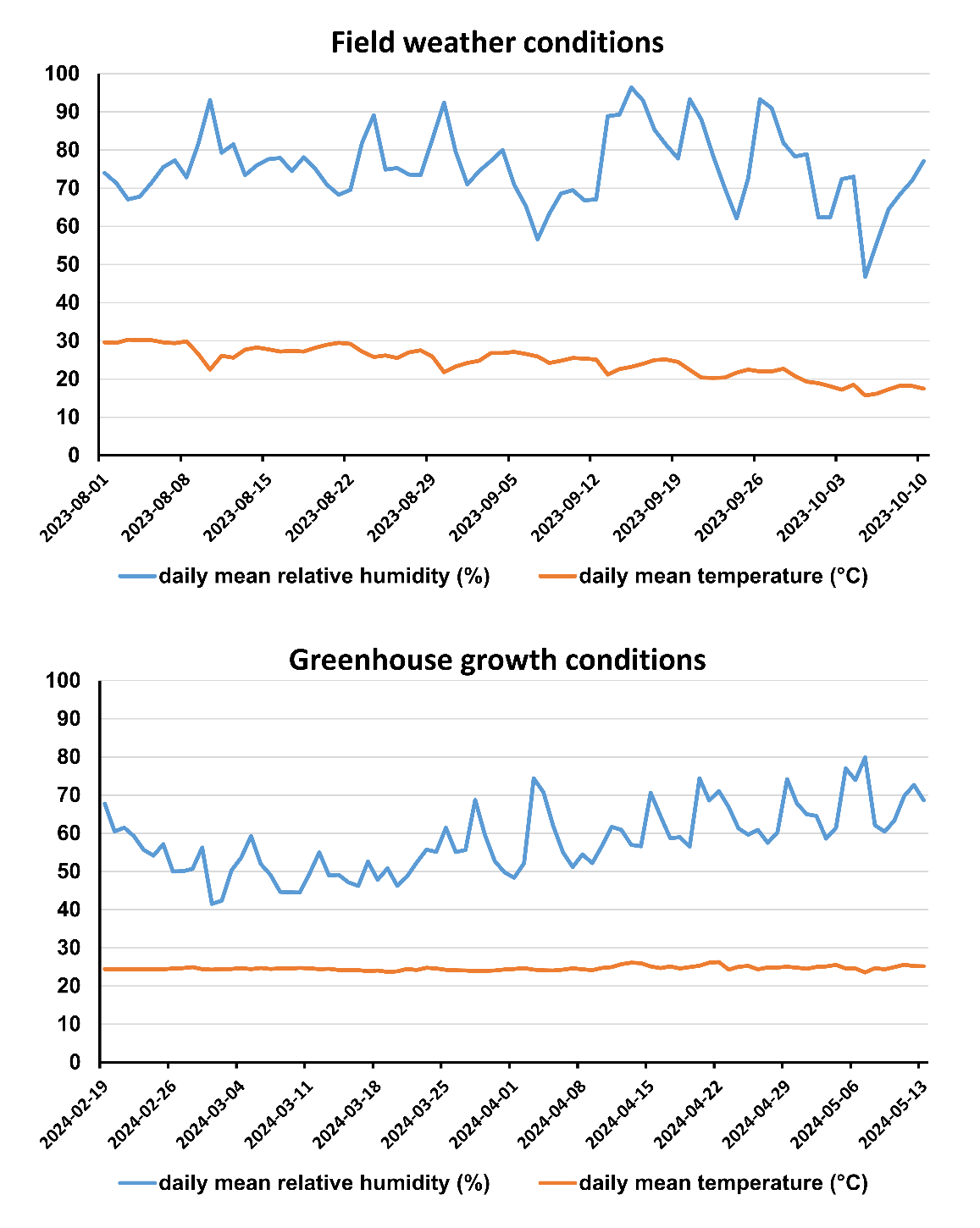


Figure S1. Weather conditions in the field (upper panel) and growth conditions in the greenhouse (lower panel) during the grain-filling stage.


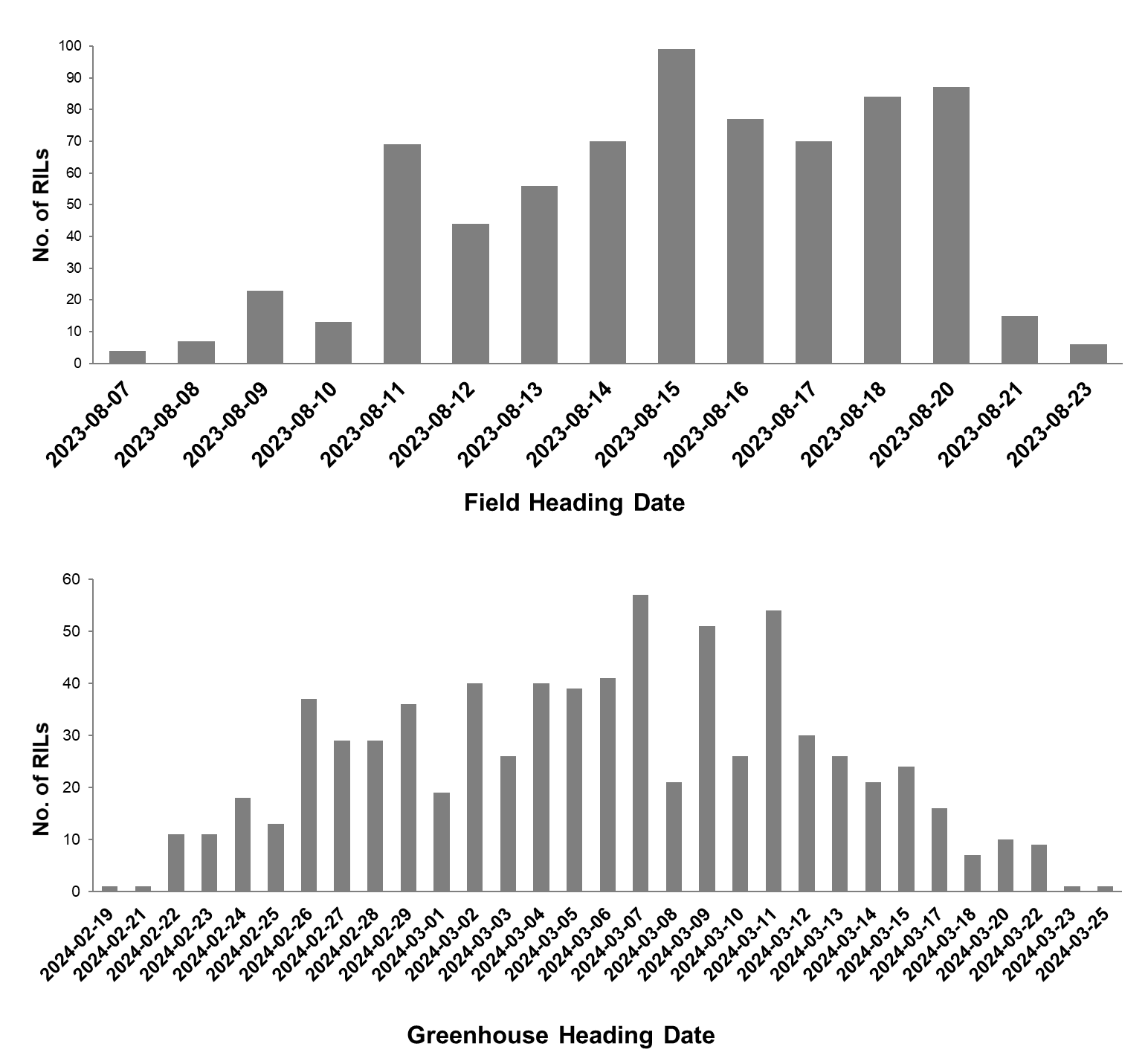


**Figure S2**. Variation in heading dates of 182 F_8_ RILs derived from a cross between Junam and Nampyeong in the field (upper panel) and greenhouse (lower panel) environments.


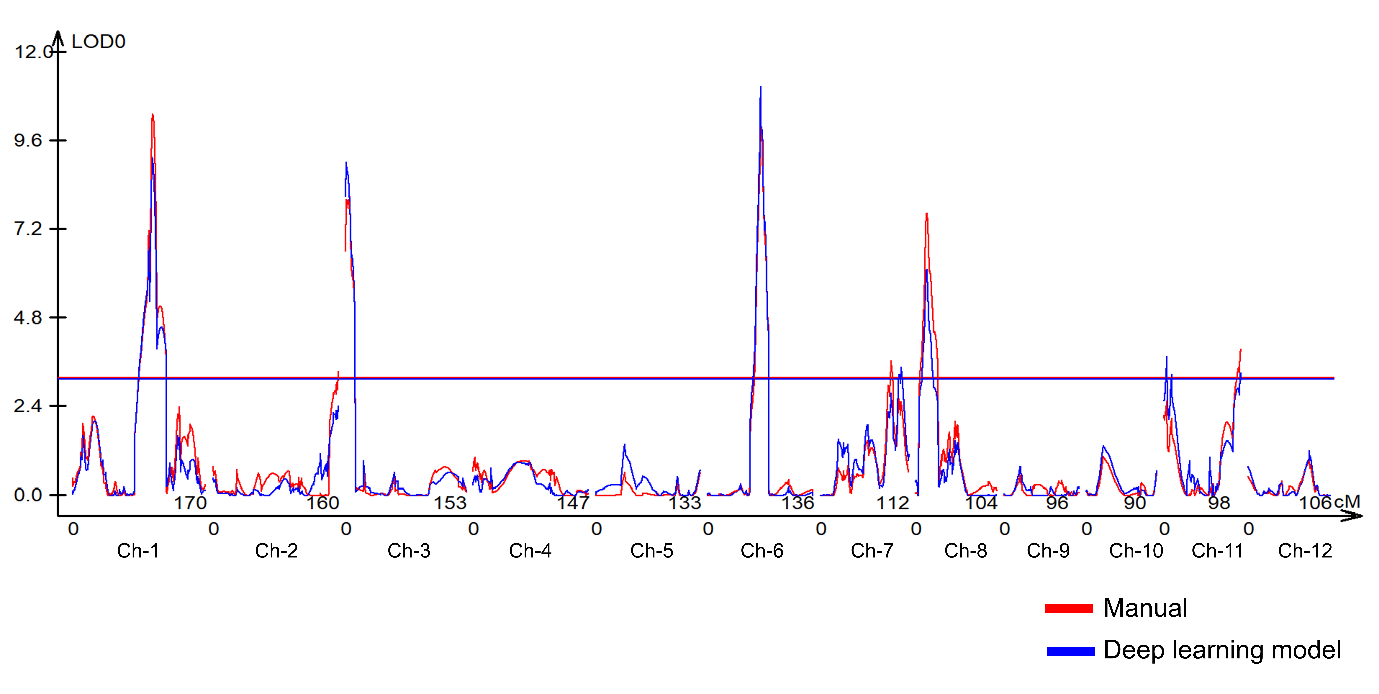


Figure S3. Comparison of QTL mapping analyses of PHS resistance under greenhouse conditions using the deep learning model (blue) and manual methods (red) of assessing the rate of PHS.


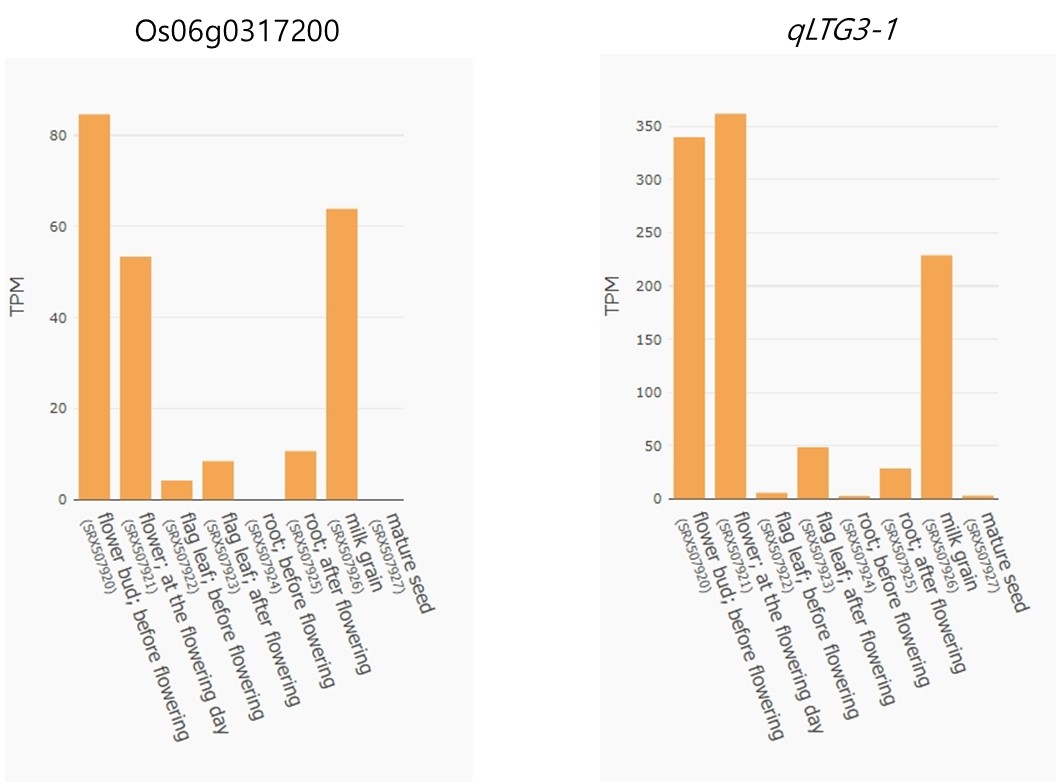


**Figure S4.** Expression profiles of *Os06g0317200* and *qLTG3-1*. These data were in silico data obtained from RAP-DB (https://rapdb.dna.affrc.go.jp/transcript/?name=Os06t0317200-02; https://rapdb.dna.affrc.go.jp/transcript/?name=Os03t0103300-01).


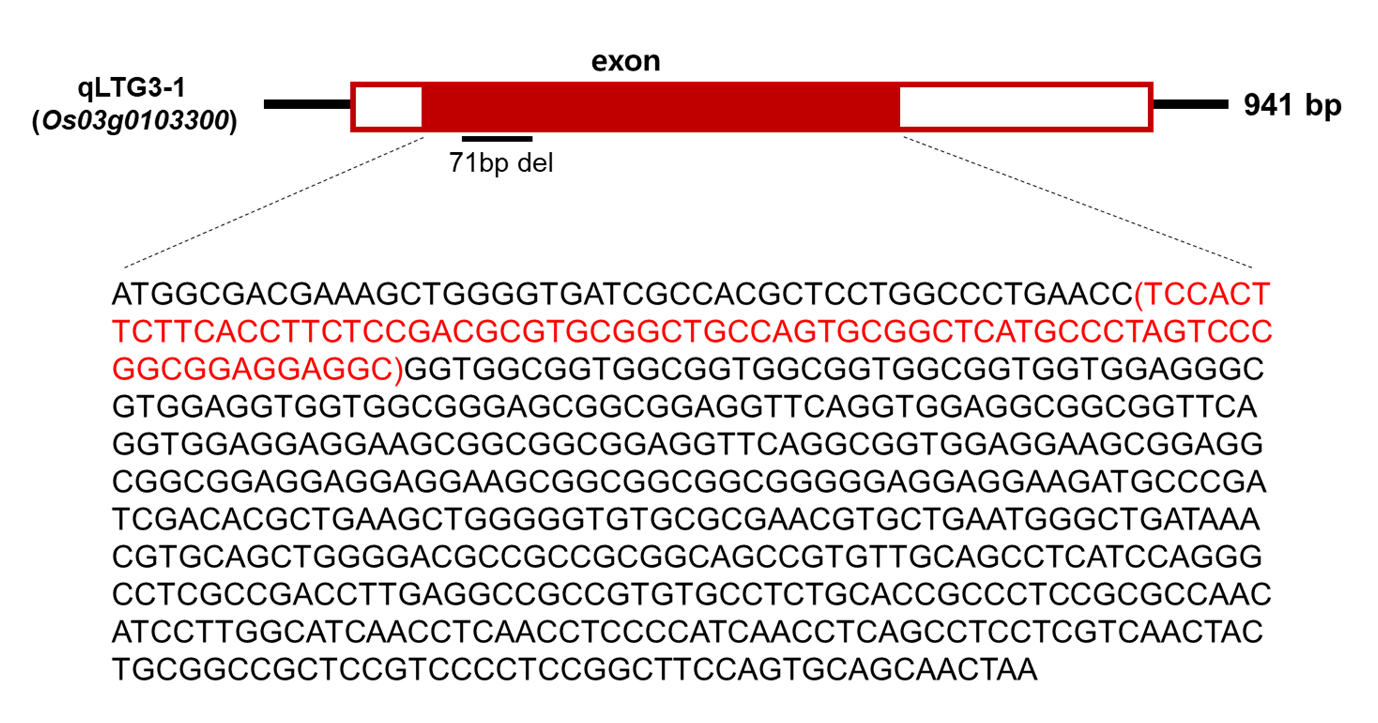


Figure S5. Structure of the *qLTG3-1* showing the 71-bp deletion in the coding region. Filled box: coding sequence (CDS); empty boxes: 5ʹ and 3ʹ untranslated regions (UTRs). The red letters in the parenthesis indicated the deleted part in Junam variety.


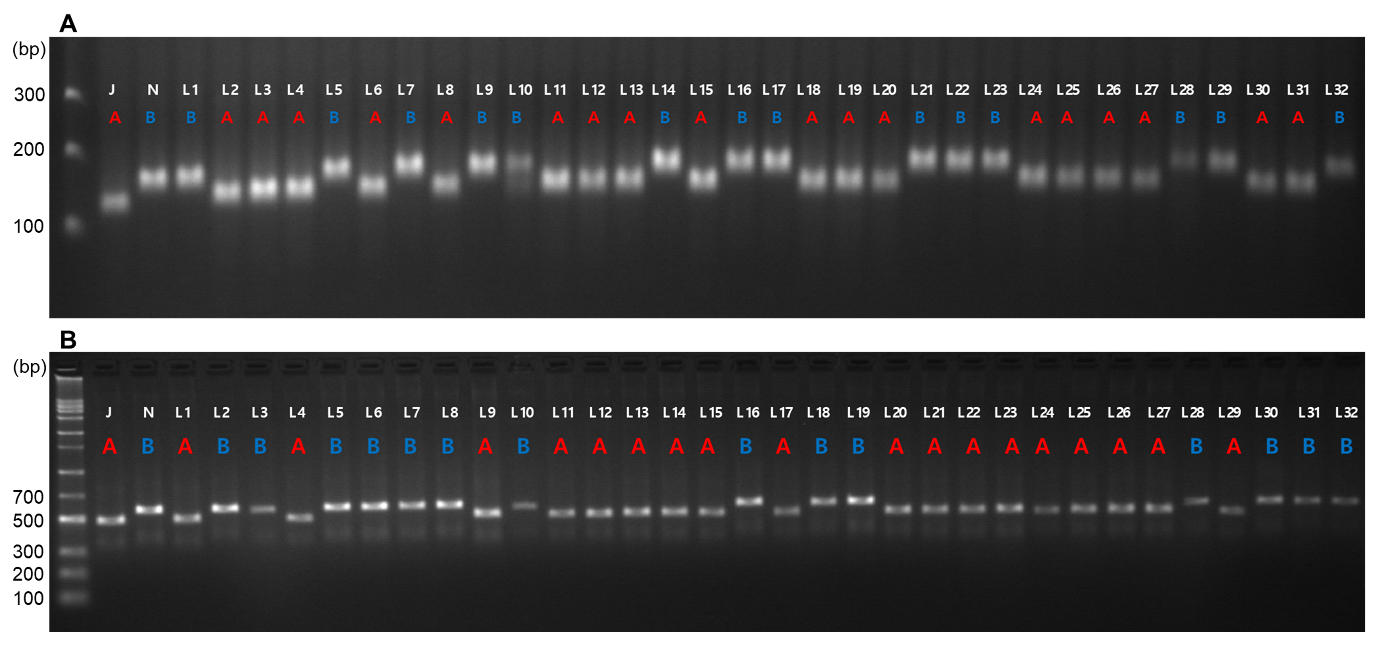


Figure S6. Segregation of genotypes in the RILs. Genotyping of RILs using the *Os06g0317200* dCAPS and *qLTG3-1* InDel markers. (A) Image of an agarose gel after electrophoresis showing RIL genotypes determined using the *Os06g0317200* dCAPS marker. J: Junam; N: Nampyeong. Digestion with *Pvu*II produced a PCR product that was 24 bp shorter in Junam than in Nampyeong. (B) Image of an agarose gel after electrophoresis showing RIL genotypes determined using the *qLTG3-1* InDel marker. The Junam PCR product was 71 bp shorter than the Nampyeong PCR product. The identification numbers of the individual RILs are shown above each lane.
